# Disruption of *Dhcr7* and *Insig1/2* in cholesterol metabolism causes defects in bone formation and homeostasis through primary cilium formation

**DOI:** 10.1038/s41413-019-0078-3

**Published:** 2020-01-02

**Authors:** Akiko Suzuki, Kenichi Ogata, Hiroki Yoshioka, Junbo Shim, Christopher A. Wassif, Forbes D. Porter, Junichi Iwata

**Affiliations:** 1 Department of Diagnostic & Biomedical Sciences, The University of Texas Health Science Center at Houston, School of Dentistry, Houston, TX USA; 2 Center for Craniofacial Research, The University of Texas Health Science Center at Houston, School of Dentistry, Houston, TX USA; 3 Division of Translational Medicine, Eunice Kennedy Shriver National Institute of Child Health and Human Development, Bethesda, MD USA; 4 Pediatric Research Center, The University of Texas Health Science Center at Houston, McGovern Medical School, Houston, TX USA; 5 MD Anderson Cancer Center UTHealth Graduate School of Biomedical Sciences, Houston, TX USA

**Keywords:** Bone, Homeostasis

## Abstract

Human linkage studies suggest that craniofacial deformities result from either genetic mutations related to cholesterol metabolism or high-cholesterol maternal diets. However, little is known about the precise roles of intracellular cholesterol metabolism in the development of craniofacial bones, the majority of which are formed through intramembranous ossification. Here, we show that an altered cholesterol metabolic status results in abnormal osteogenesis through dysregulation of primary cilium formation during bone formation. We found that cholesterol metabolic aberrations, induced through disruption of either *Dhcr7* (which encodes an enzyme involved in cholesterol synthesis) or *Insig1* and *Insig2* (which provide a negative feedback mechanism for cholesterol biosynthesis), result in osteoblast differentiation abnormalities. Notably, the primary cilia responsible for sensing extracellular cues were altered in number and length through dysregulated ciliary vesicle fusion in *Dhcr7* and *Insig1/2* mutant osteoblasts. As a consequence, WNT/β-catenin and hedgehog signaling activities were altered through dysregulated primary cilium formation. Strikingly, the normalization of defective cholesterol metabolism by simvastatin, a drug used in the treatment of cholesterol metabolic aberrations, rescued the abnormalities in both ciliogenesis and osteogenesis in vitro and in vivo. Thus, our results indicate that proper intracellular cholesterol status is crucial for primary cilium formation during skull formation and homeostasis.

## Introduction

Cellular cholesterol amounts are regulated by multiple steps of biosynthesis and feedback mechanisms.^1^ Infants with cholesterol metabolism aberrations have malformations in the craniofacial region.^2–5^ For example, mutations in genes involved in cholesterol synthesis (*DHCR7*, *SC5D*, and *DHCR24*) have been found in patients with Smith-Lemli-Opitz Syndrome (SLOS) lathosterolosis, and desmosterolosis, who display craniofacial bone abnormalities.^1^ In addition, high-cholesterol diets during pregnancy are known to be a risk factor for birth defects, including craniofacial bone abnormalities.^6,7^ Despite these findings, it remains unclear how cholesterol metabolism contributes to craniofacial bone formation, particularly intramembranous ossification.

The 7-dehydrocholesterol reductase (DHCR7) catalyzes the final step of cholesterol biosynthesis;^8^ mutations in *DHCR7* cause cholesterol deficiency and an excess of cholesterol precursors, resulting in craniofacial deformities (e.g., microcephaly, cleft palate, craniosynostosis, and micrognathia), intellectual disability, and behavioral problems in humans.^9,10^ *Dhcr7*^*−/−*^ mice show a suckling defect, weight less, immature lungs, distended bladders, and variable craniofacial abnormalities.^11^ The molecular mechanism of craniofacial anomalies in these conditions is still elusive. The insulin-induced genes 1 and 2 (INSIG1 and INSIG2) are endoplasmic reticulum (ER) retention proteins that play roles in both the regulation of the activity of the 3-hydroxy-3-methylglutaryl-coenzyme A (HMG-CoA) reductase and the translocation of the sterol regulatory element-binding protein (SREBP) to the nucleus for gene regulation.^12^ Mice deficient for *Insig1* and *Insig2* (*Insig1*^*−/−*^*;Insig2*^*−/−*^ mice), which are negative regulators of cholesterol biosynthesis,^13^ show high-cholesterol levels in craniofacial tissues and display craniofacial deformities such as midfacial cleft, cleft palate, calvarial deformities and micrognathia, while mice deficient for either *Insig1* or *Insig2* are normal.^3,12^ These craniofacial deformities are rescued by the normalization of cholesterol levels in *Insig1/2* null mice;^3^ however, it remains elusive how high-cholesterol levels cause craniofacial deformities and which cells are responsible for the craniofacial anomalies seen in *Insig1/2* null mice.

Primary cilia, microtubule-based organelles that function in sensory and signaling pathways, are enriched with cholesterol-rich microdomains (known as lipid rafts) that recruit or retain receptors and ciliary membrane proteins.^14^ An association between lipid rafts and ciliary membrane proteins has been suggested in other organisms, including vertebrate photoreceptors,^15^ *Chlamydomonas reinhardtii*,^16^ mammalian spermatozoa,^17^ and *Leishmania major*.^18^ Defects of primary cilia cause various deformities, including craniofacial abnormalities (altogether known as ciliopathies, a group of genetic syndromes associated with defects in primary cilia).^19,20^ The broad-spectrum phenotypes in *Dhcr7*^*−/−*^ mice and individuals with SLOS^11^ (e.g., presenting with craniofacial anomalies such as craniosynostosis, hypertelorism, and cleft palate, as well as immature lungs and enlarged bladders) are similar to those seen in ciliopathies. The phenotypic similarity between ciliopathies and cholesterol synthesis defects suggests that cholesterol metabolism (level and function of mature cholesterol and cholesterol intermediates) can regulate bone development through modulation of primary cilium formation and function. While over the past decade the underlying mechanism of ciliopathies has focused on the inner structures of primary cilia such as intraflagellar transport (IFT) and kinesin (KIF) proteins,^21^ little is known about the role of the surface membrane characteristics of primary cilia in ciliogenesis.

In this study, we investigated the link between cholesterol metabolic aberrations and craniofacial bone abnormalities by employing both loss-of-function and gain-of-function mouse models: mice with a deletion of *Dhcr7* and mice with a deletion of *Insig1/2*, respectively. To identify the bone abnormalities with either low or high-cholesterol levels, we carried out microCT, skeletal staining, and histological analyses in these mice. Our study aims to elucidate how *Dhcr7* and *Insig1/2* regulate bone formation.

## Results

### *Dhcr7* deficiency increases osteogenesis

**Fig. 1:**
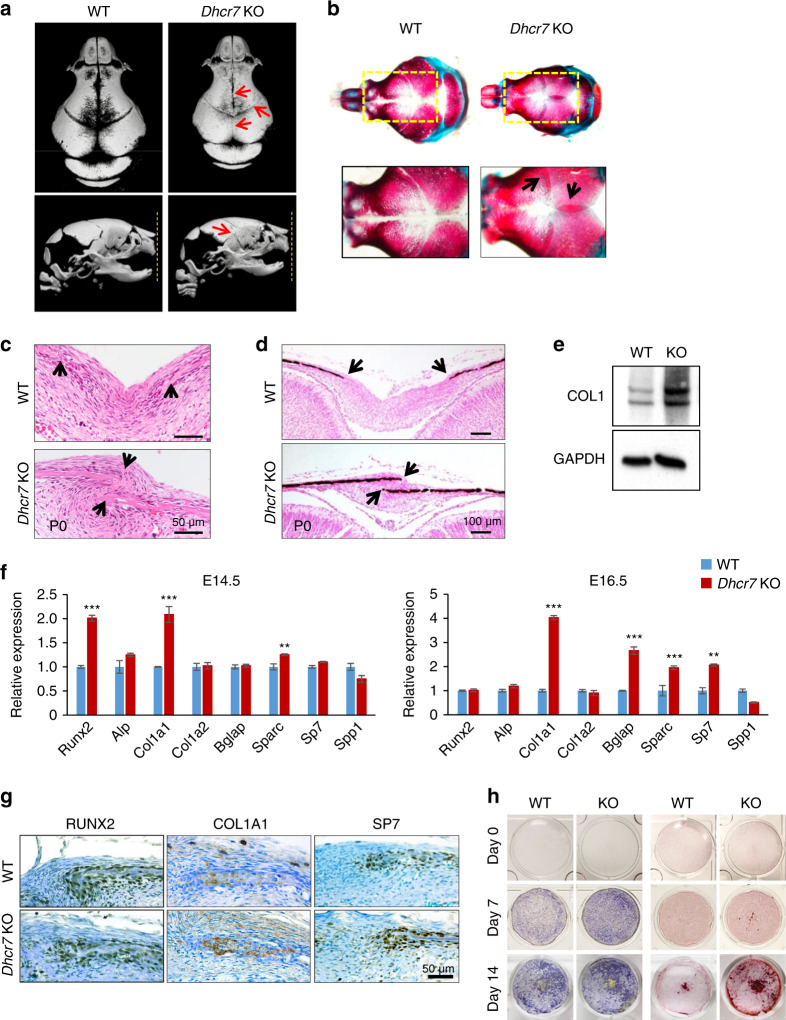
Loss of *Dhcr7* accelerates osteogenesis. **a** MicroCT images of the calvaria—top view (upper panels) and side view (lower panels)—of newborn wild-type (WT) control and *Dhcr7*^*−/−*^ knockout (KO) mice. Red arrows indicate early closure of the metopic, coronal, and sagittal sutures. Yellow dotted lines in the lower panels indicate the tips of premaxillae. **b** Skeletal staining of skulls from newborn WT control and KO mice. Yellow boxed areas are enlarged in the lower images. Black arrows in KO images indicate overlapping of frontal and parietal bones at the coronal suture and left and right parietal bones at the sagittal suture. **c** Hematoxylin and Eosin (H&E) staining of the sagittal sutures of newborn WT and KO mice. Arrows indicate the osteogenic front. Scale bar, 50 µm. **d** von Kossa staining of the sagittal sutures of newborn WT and KO mice. Arrows indicate the osteogenic front. Scale bar, 100 µm. **e** Immunoblotting for type I collagen (COL1) in P0 calvaria of WT and KO mice. GADPH was used as loading control. **f** Quantitative RT-PCR of the indicated osteogenic genes at E14.5 (left) and E16.5 (right) in WT (blue bars) and KO (red bars) mice. *n* = 6 per genotype per stage. **P < 0.01; ***P < 0.001. **g** Immunohistochemistry analysis for RUNX2, COL1A1 and SP7 (Osterix) in newborn WT and KO mice. Nuclei were counterstained with 0.04% methylene blue. Scale bar, 50 µm. **h** Alkaline phosphatase (left) and Alizarin Red (right) staining of osteoblasts isolated from newborn WT and KO calvaria after induction of osteogenic differentiation at Day 0, 7, and 14.

*Dhcr7*^*−/−*^ knockout (KO) mice presented microcephaly, accelerated bone formation, and thicker calvaria bones at birth with complete penetrance, and died within 1 day after birth (Fig. 1a–c and Supplementary Fig. S1). The accelerated bone formation resulted in immature suture fusion after culturing calvaria explants for 3 days (Supplementary Fig. S1d). To examine the cellular mechanism of how cholesterol metabolic aberrations cause accelerated bone formation in *Dhcr7*^*−/−*^ mice, we carried out biological analyses, namely BrdU incorporation assays and Ki67 immunohistochemistry for cell proliferation, TUNEL assays for apoptosis, and von Kossa staining for mineralization, and immunoblotting for type I collagen for osteogenic differentiation. We found that osteogenic differentiation, but not cell proliferation and apoptosis, was increased in *Dhcr7*^*−/−*^ frontal bones (Fig. 1d, e and Supplementary Fig. S2). Next, to determine the regulatory mechanism of osteogenic differentiation, we performed quantitative RT-PCR (qRT-PCR) analyses for osteogenic factors (*Runx2*, *Alp*, *Col1a1*, *Col1a2*, *Bglap*, *Sparc*, *Sp7*, and *Spp1*) using frontal bones from *Dhcr7*^*−/−*^ mice at embryonic day (E) E14.5, E15.5, E16.5, and postnatal day (P) P0 (Fig. 1f and Supplementary Fig. S3). *Col1a1* gene expression was significantly and consistently upregulated in *Dhcr7*^*−/−*^ frontal bones compared to controls at E14.5-P0. Expression of RUNX2, COL1A1 and SP7 (*aka* Osterix) was increased, and the area with these positive signals was expanded compared to controls and correlated with increased expression of these genes in *Dhcr7*^*−/−*^ frontal bones (Fig. 1f, g). Next, we evaluated the effect of loss of *Dhcr7* on osteogenic differentiation using cultured osteoblasts from P0 frontal bones. The *Dhcr7*^*−/−*^ osteoblast characteristics (no proliferation defect but accelerated osteogenic differentiation through upregulated *Col1a1* expression) were well conserved in cultured primary osteoblasts (Fig. 1h and Supplementary Fig. S3). Taken together, our results indicate that a failure in cholesterol synthesis causes accelerated osteogenesis through upregulated *Col1a1* expression.

### *Insig1/2* deficiency suppresses osteogenesis

**Fig. 2:**
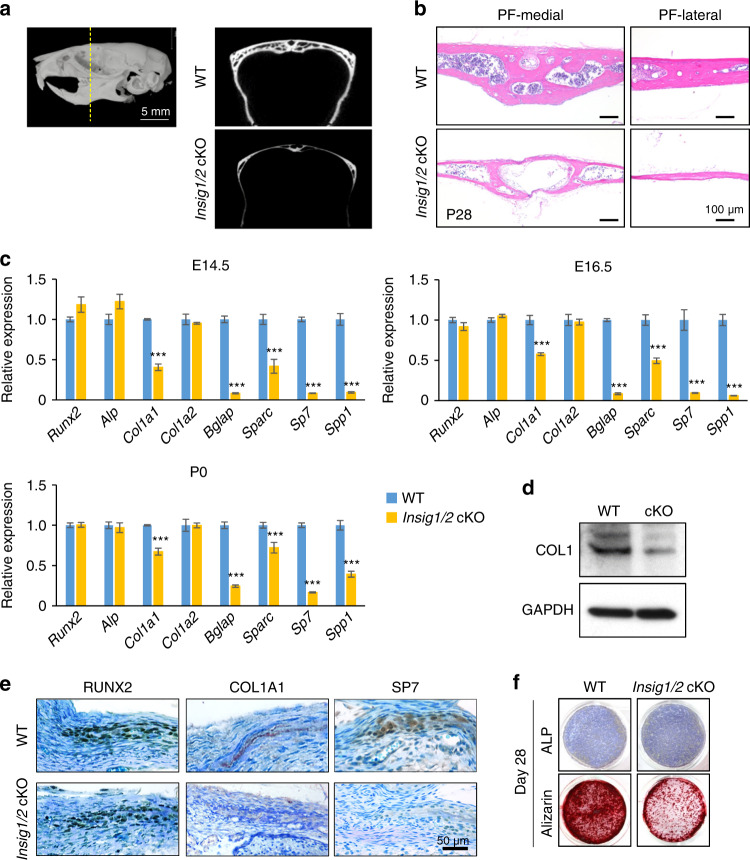
Excessive cholesterol synthesis decreased bone formation in *Insig1/2* cKO mice. **a** MicroCT images of the frontal bones (a slice at the level of the distal end of the 3rd molar, indicated by the yellow dotted line) of WT and *Insig1/2* conditional KO (cKO) mice at P35. **b** H&E staining of the medial and lateral regions of the posterior frontal bones (PF) from P28 WT and *Insig1/2* cKO mice. Scale bars, 100 µm. **c** Quantitative RT-PCR for the indicated osteogenic genes in WT (blue bars) and *Insig1/2* cKO (yellow bars) mice at E14.5, E16.5, and P0. *n* = 6 per genotype per stage. ***P < 0.001. **d** Immunoblotting for COL1 in newborn WT and *Insig1/2* cKO mice. GADPH was used as loading control. **e** Immunohistochemical analysis for RUNX2, COL1A1 and SP7 (Osterix) in newborn WT and *Insig1/2* cKO mice. Nuclei were counterstained with 0.04% methylene blue. Scale bar, 50 µm. **f** Alkaline phosphatase (top) and Alizarin Red (bottom) staining of osteoblasts isolated from the frontal bones of newborn WT and *Insig1/2* cKO after induction of osteogenic differentiation at Day 28.

To determine the tissue-specific contribution of high-cholesterol status to craniofacial deformities, we employed *Insig1*/*2* conditional knockout (cKO) mice^12^ in cranial neural crest (CNC) cells^22^, which give origin to the majority of craniofacial bones.^23^ *Insig1/2* cKO mice were viable, but they exhibited very thin frontal bones at birth and later in life (Fig. 2a, b), while the other craniofacial structures derived from CNC cells in these mice were intact. To examine the cellular mechanism of how high-cholesterol amount results in decreased bone formation in *Insig1/2* cKO mice, we carried out biological analyses, as performed in *Dhcr7*^*−/−*^ mice. We found that osteogenic differentiation, but not cell proliferation and apoptosis, was decreased in *Insig1/2* cKO frontal bones (Supplementary Fig. S4). We also found that *Col1a1* gene expression was significantly and consistently downregulated in frontal bones of *Insig1/2* cKO mice at E14.5, E16.5 and P0 (Fig. 2c), with protein expression correlating with gene expression (Fig. 2d). Expression of COL1A1 and SP7, but not RUNX2, was decreased, which was correlated with the expressions of these genes in *Insig1/2* cKO frontal bones (Fig. 2e). We further evaluated the effect of loss of *Insig1/2* on osteogenic differentiation using cultured osteoblasts from *Insig1/2* cKO mice (Fig. 2f and Supplementary Fig. S4). The *Insig1/2* cKO osteoblast characteristics (no proliferation defect but reduced osteogenic differentiation through decreased *Col1a1* expression) were well conserved in cultured primary osteoblasts. Since COL1 expression was decreased in *Insig1/2* cKO mice, the abnormalities may resemble osteogenesis imperfecta, which is a congenital bone disorder characterized by thinner and fragile bones that affects 6–7 in 100 000 individuals worldwide.^24^ Altogether, our results indicate that either too much or too little cholesterol causes calvarial bone abnormalities through dysregulation of *Col1a1* expression.

### Ciliogenesis is altered in either *Dhcr7*- or *Insig1/2*-deficient mice

**Fig. 3:**
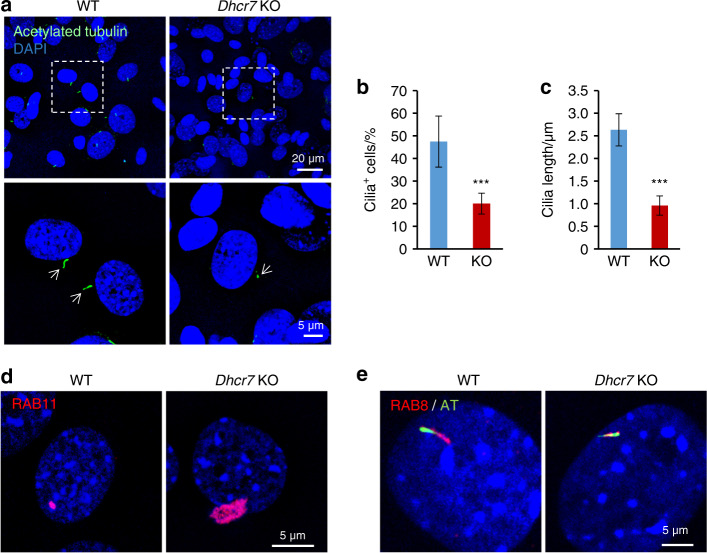
Impaired cholesterol synthesis results in defective ciliogenesis in *Dhcr7* KO osteoblasts. **a** Immunocytochemistry (IC) analyses of primary cilia in osteoblasts from wild-type (WT) control and *Dhcr7* knockout (KO) mice. Primary cilia were stained with anti-acetylated tubulin antibody (green), and nuclei were stained with DAPI (blue). Boxed areas in upper images are enlarged, and arrows indicate primary cilia. Scale bars: 20 µm in the upper images and 5 µm in the lower images. Arrows indicate primary cilia. **b** Percentage of cells with primary cilia in osteoblasts from WT (blue bar) and *Dhcr7* KO (red bar) mice. More than 200 cells were randomly analyzed in three independent experiments. ***P < 0.001. **c** Quantifi**c**ation of the length of primary cilia in osteoblasts from WT (blue bar) and *Dhcr7* KO (red bar) mice. More than 200 cells were randomly analyzed in three independent experiments. ***P < 0.001. **d** IC analyses for RAB11 (red) in WT and *Dhcr7* KO osteoblasts. Nuclei were stained with DAPI (blue). Scale bar, 5 µm. **e** IC analyses for RAB8 (red) and acetylated tubulin (AT; green) in WT and *Dhcr7* KO osteoblasts. Nuclei were stained with DAPI (blue). Scale bar, 5 µm.

As cholesterol is abundant in cellular membranes, we carefully investigated cellular membranous structures and found that there were fewer and shorter primary cilia in *Dhcr7*^*−/−*^ osteoblasts compared to controls (Fig. 3a–c). Ciliogenesis starts with the interaction of the basal body (mother centriole) with primary ciliary vesicles (CVs), which can be labeled with RAB11,^25^ and then the axoneme grows within the ciliary membrane while fusing with secondary CVs, which can be labeled with RAB8.^26^ The elongated primary cilium eventually fuses with the plasma membrane, allowing the distal part of the cilium to interact with the extracellular milieu.^19,27^ To track ciliogenesis in *Dhcr7*^*−/−*^ osteoblasts, CVs were immunostained with anti-RAB11 and anti-RAB8 antibodies. We found that RAB11-positive primary CVs accumulated in the cells and that RAB8-positive secondary CVs failed to gather at sites of cilium formation in *Dhcr7*^*−/−*^ osteoblasts (Fig. 3d, e).

**Fig. 4:**
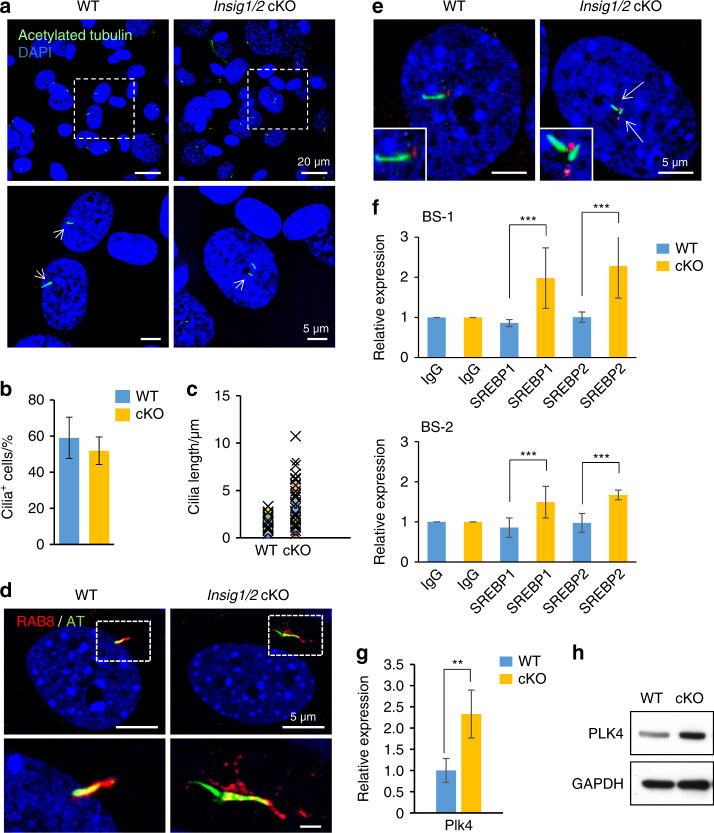
Excessive cholesterol synthesis results in abnormal primary cilium formation. **a** IC analyses for anti-acetylated tubulin (AT; green) in WT and *Insig1/2* conditional KO (cKO) osteoblasts. Nuclei were stained with DAPI (blue). Boxed areas in upper images are enlarged, and arrows indicate primary cilia. Scale bars: 20 µm in the upper images and 5 µm in the lower images. **b** Percentage of cells with primary cilia in osteoblasts from WT (blue bar) and *Insig1/2* cKO (yellow bar) osteoblasts. More than 200 cells were randomly analyzed in three independent experiments. **c** Quantification of the length of primary cilia in osteoblasts from WT (left) and *Insig1/2* cKO (right) mice. More than 200 cells were randomly analyzed in three independent experiments. ***P < 0.001. **d** IC for RAB8 (red) and AT (green) in WT and *Insig1/2* cKO osteoblasts. Nuclei were stained with DAPI (blue). Boxed areas in upper images are enlarged. Scale bars, 5 µm. **e** IC analyses for γ-tubulin (red) and AT (green) in WT and *Insig1/2* cKO osteoblasts. Nuclei were stained with DAPI (blue). Arrows indicate duplicated primary cilia and basal bodies. Primary cilia are enlarged in insets. Scale bars, 5 µm. **f** ChIP assays of IgG control and SREBP1 or SREBP2 for SRE (BS1 and BS2) of the *Plk4* promoter region in WT control (blue bars) and *Insig1/2* cKO (yellow bars) osteoblasts. *n* = 3 per group. ***P < 0.001. **g** Quantitative RT-PCR for *Plk4* in WT (blue bar) and *Insig1/2* cKO (yellow bar) osteoblasts. *n* = 6 per group. **P < 0.01. **h** Immunoblotting for PLK4 in WT and *Insig1/2* cKO osteoblasts. GAPDH was used as loading control.

On the other hand, *Insig1/2* cKO osteoblasts displayed multiple and longer primary cilia compared to controls (Fig. 4a–c), and RAB8-positive CVs were abnormally fused with each other (Fig. 4d). The osteogenic and primary cilium phenotypes in *Insig1/2* cKO osteoblasts were recapitulated with transfection of an adenovirus-Cre system (Supplementary Fig. S5). In addition, multiple basal bodies were detected, consistent with the presence of multiple primary cilia in *Insig1/2* cKO osteoblasts (Fig. 4e and Supplementary Figs. S5 and S6). We then hypothesized that gene expression of molecules involved in the formation of the pericentriolar material (PCM) complex (i.e., *Atf5*, *Aurka*, *Bbs4*, *Cdk5rap2*, *Cep152*, *Cep192*, *Cpap*, *Lck*, *Nin*, *Pcm1*, *Pcnt*, *Plk1*, *Plk4, Sass6, Stil*, *Tube1*, *Tubg1*, *Tunks*, and *Tunks2*), which is important for proper centriole formation, was altered in *Insig1/2* cKO osteoblasts. Previous studies indicate that overexpression of *Plk1*, *Plk4*, *Stil* and *Sass6* induces the formation of multiple centrioles.^28–35^ Among them, by conducting a bioinformatics promoter analysis we found that the *Plk4* promoter contained two potential sterol regulatory elements (SREs) for the SRE-binding protein (SREBP) binding. To access the binding experimentally, we carried out chromatin immunoprecipitation (ChIP) assays for the sites [site 1; GTGGAGAGT (-244 bp to -252 bp) and site 2; TCACTCAGC (–1295 bp to –1303 bp)] in *Insig1/2* cKO and control osteoblasts and found increased binding of SREBP1 and SREBP2 to the SREs in *Insig1/2* cKO osteoblasts (Fig. 4f). Indeed, expression of the *Plk4* gene was significantly and specifically upregulated in *Insig1/2* cKO osteoblasts compared to controls (Fig. 4g), while the expression of other PCM-related genes was not altered (Supplementary Fig. S6b). We further confirmed that PLK4 protein expression was increased in the mutant osteoblasts compared to controls (Fig. 4h). Taken together, our results indicate that *Dhcr7* and *Insig1/2* play an important role in primary cilium formation.

WNT/β-catenin and hedgehog signaling pathways are downstream signaling cascades responsible for the bone phenotype of *Dhcr7*- and *Insig1/2*-deficient mice

**Fig. 5:**
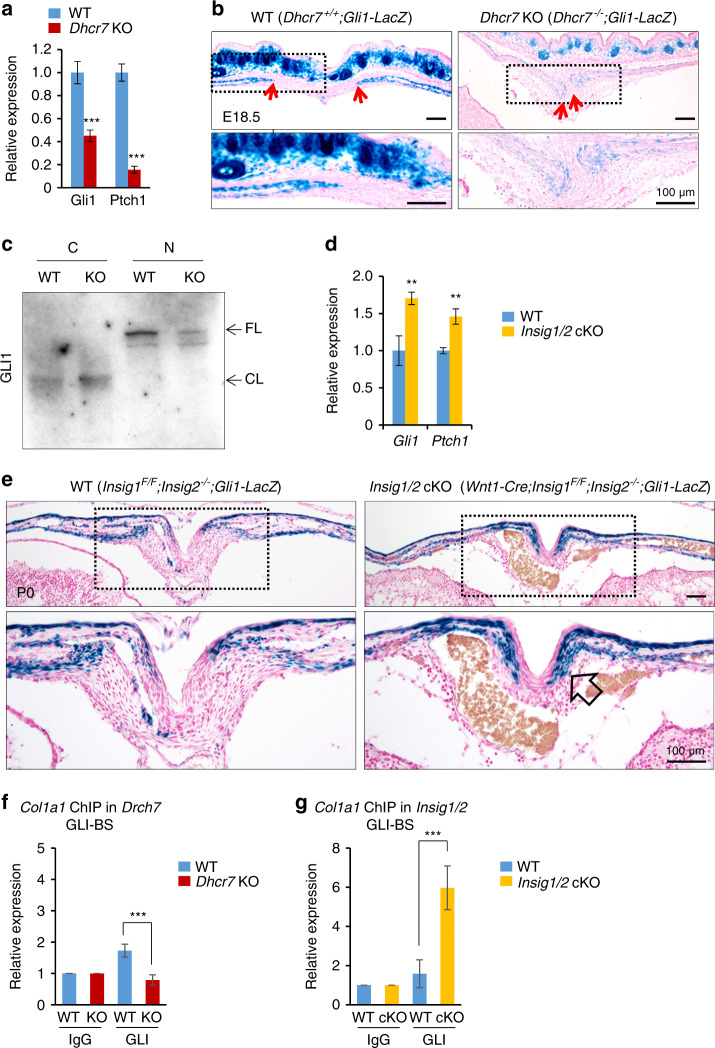
Altered hedgehog signaling in calvaria from *Dhcr7* and *Insig1/2* mutant mice during craniofacial development. **a** Quantitative RT-PCR analyses for *Gli1* and *Ptch1* expression as a readout for HH signaling activity in newborn wild-type (WT) control (blue bars) and *Dhcr7* knockout (KO; red bars) calvaria. *n* = 6 per group. ***P < 0.001. **b** β-galactosidase staining (blue) for sites of HH signaling activation in the frontal bones of E18.5 *Dhcr7*^*+/+*^*;Gli1-LacZ* (WT) and *Dhcr7*^*−/−*^*;Gli1-LacZ* (*Dhcr7* KO) mice. Red arrows indicate osteogenic fronts; boxed areas are enlarged in lower images. Nuclei were stained with nuclear fast red. Scale bars, 100 µm. **c** Cell fra**c**tionation and subsequent immunoblotting analysis for GLI1, using cytosol (C) and nuclear (N) fractions from WT control and *Dhcr7* KO osteoblasts. FL, full-length; CL, cleaved. **d** Quantitative RT-PCR analysis for *Gli1* and *Ptch1* in newborn WT control (blue bars) and *Insig1/2* conditional KO (cKO; yellow bars) frontal bones. *n* = 6 per group. **P < 0.01. **e** β-galactosidase staining (blue) for sites of HH signaling activation in the frontal bones of P0 *Insig1*^*F/F*^*;Insig2*^*−/−*^*;Gli1-LacZ* (WT) and *Wnt1-Cre;Insig1*^*F/F*^*;Insig2*^*−/−*^*;Gli1-LacZ* (*Insig1/2* cKO) mice. Open arrow indicates increased Gli1-LacZ; boxed areas are enlarged in lower images. Nuclei were stained with nuclear fast red. Scale bars, 100 µm. **f** ChIP assays of IgG control and GLI-1 for the *Col1a1* promoter region in *Dhcr7* KO (red bars) and WT control (blue bars) osteoblasts. *n* = 3 per group. ***P < 0.001. **g** ChIP assays of IgG control and GLI-1 for the *Col1a1* promoter region in *Insig1/2* cKO (yellow bars) and WT control (blue bars) osteoblasts. *n* = 3 per group. ***P < 0.001.

We examined how the ciliary phenotypes resulted in osteogenic abnormalities. The primary cilia coordinate with multiple signaling pathways such as hedgehog (HH) and WNT during osteogenesis.^36–38^ To investigate HH signaling in *Dhcr7*^*−/−*^ calvaria, we analyzed *Gli1* and *Ptch1* levels by qRT-PCR, which are readout gene expressions for HH signaling activity.^39–43^ We found that expression of these genes was significantly downregulated in *Dhcr7*^*−/−*^ calvaria (Fig. 5a). Furthermore, we employed *Gli1-LacZ* reporter mice to evaluate the HH signaling activity in vivo. We found that HH signaling was compromised in the calvaria of *Dhcr7*^*−/−*^ mice compared to controls (Fig. 5b). To further confirm the reduced HH signaling activity in the calvaria of *Dhcr7*^*−/−*^ mice, we performed cell fractionation and the consequent immunoblotting analyses for GLI1. Previous studies indicate that in the absence of HH ligands, GLI1 is truncated and degraded by the proteasome in the cytoplasm, while GLI1 translocates into the nucleus in the presence of HH ligands by escaping from degradation.^44,45^ As expected, full-length GLI1 was decreased in the nuclear fraction from *Dhcr7*^*−/−*^ osteoblasts (Fig. 5c).

Next, we investigated the HH signaling activity in *Insig1/2* cKO mice. We carried out qRT-PCR for *Gli1* and *Ptch1* in *Insig1/2* cKO calvaria and found that expression of these genes was significantly upregulated in *Insig1/2* cKO mice (Fig. 5d). Furthermore, we performed LacZ staining for *Gli1* expression in *Insig1/2 cKO;Gli1-LacZ* and control mice. As expected, *Gli1-LacZ* expression was increased in the calvaria of *Insig1/2 cKO;Gli1-LacZ* mice compared to that of control mice (Fig. 5e). Next, to examine whether *Col1a1* expression was regulated through HH signaling, we conducted promoter analyses for GLI binding on the *Col1a1* promoter and found that the *Col1a1* promoter contained a putative GLI-binding site (GGCCACGCA; −68 bp to –60 bp) (Supplementary Fig. S7a). ChIP assays validated that GLI binding to the *Col1a1* promoter region was correlated with the activity of HH signaling in osteoblasts from *Dhcr7*^*−/−*^ and *Insig1/2* cKO mice (Fig. 5f, g).

Thus, HH signaling activity was correlated with the primary cilium phenotype of *Dhcr7*^*−/−*^ and *Insig1/2* cKO mice. However, previous studies show that upregulated and downregulated HH signaling cause defective and normal craniofacial bone formation, respectively.^46–48^ This suggests that altered HH signaling was not responsible for the bone phenotypes in *Dhcr7*^*−/−*^ and *Insig1/2* cKO mice.

**Fig. 6:**
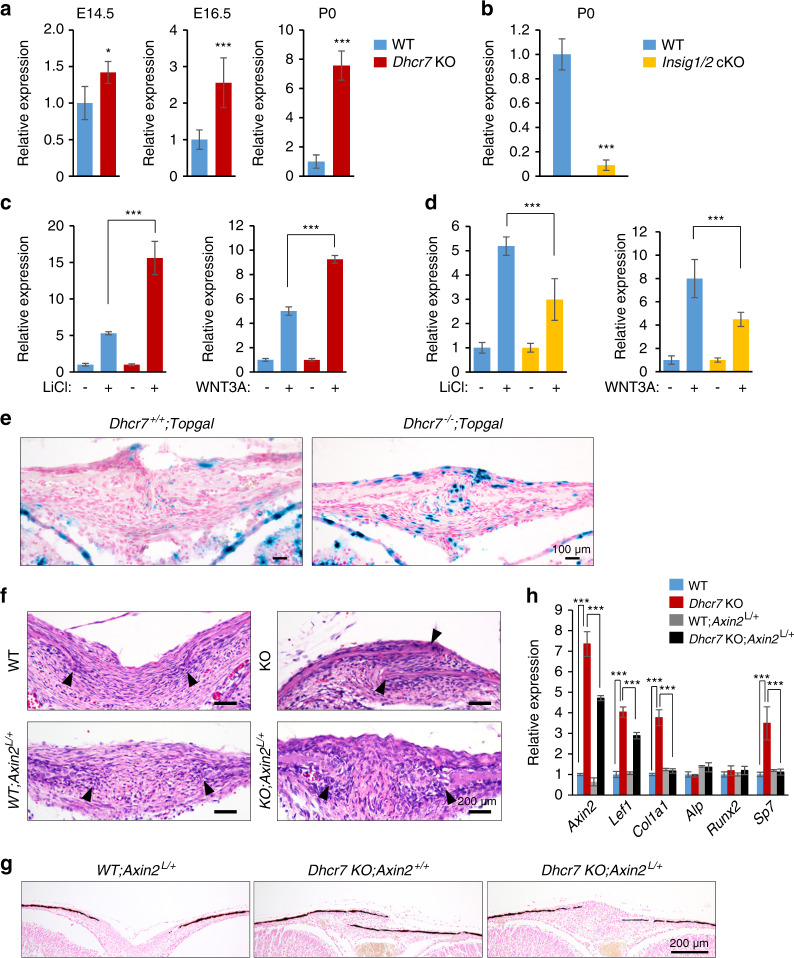
Altered WNT/β-catenin signaling in calvaria from *Dhcr7* and *Insig1/2* mutant mice during craniofacial development. **a** Quantitative RT-PCR for *Axin2* in calvaria from wild-type (WT; blue bars) control and *Dhcr7* knockout (KO; red bars) mice at E14.5, E16.5, and P0 (newborn). *n* = 6 per group. *P < 0.05; ***p < 0.001. **b** Quantitative RT-PCR for *Axin2* in calvaria from newborn WT (blue bar) and *Insig1/2* conditional KO (cKO; yellow bar) mice. *n* = 6 per group. ***P < 0.001. **c** Quantitative RT-PCR for *Axin2* after treatment with LiCl (left panel) or WNT3A (right panel) in WT (blue bars) and *Dhcr7* KO (red bars) osteoblasts. *n* = 6 per group. ***P *<* 0.001. **d** Quantitative RT-PCR for *Axin2* after treatment with LiCl (left panel) or WNT3A (right panel) in WT (blue bars) and *Insig1/2* cKO (yellow bars) osteoblasts. *n* = 6 per group. ***P < 0.001. **e** β-galactosidase staining (blue) for sites of WNT/β-catenin signaling activation in the frontal bones of P0 *Dhcr7*^*+/+*^*;Topgal* and *Dhcr7*^*−/−*^*;Topgal* mice. Nuclei were stained with nuclear fast red. Scale bars, 100 µm. **f** Hematoxylin and Eosin staining of the sagittal sutures of newborn *WT*, *WT;Axin2*^*L/+*^, *Dhcr7 KO* and *Dhcr7 KO;Axin2*^*L/+*^ mice. Arrowheads indicate the osteogenic front. Accelerated bone formation of the sutures (frontal, coronal, and sagittal sutures) was normalized in newborn *Dhcr7 KO;Axin2*^*L/+*^ mice (*n* = 6/6). Scale bars, 100 µm. **g** Von Kossa staining of the sagittal sutures of newborn *WT;Axin2*^*L/+*^, *Dhcr7 KO* and *Dhcr7 KO;Axin2*^*L/+*^ mice. Scale bar, 200 µm. **h** Quantitative RT-PCR of the indicated genes in newborn WT (blue bars), *Dhcr7* KO (red bars), *WT;Axin2*^*L/+*^ (gray bars) and *Dhcr7KO;Axin2*^*L/+*^ (black bars) mice. *n* = 6 per group. ***P < 0.001.

We, therefore, investigated another candidate cilium-mediated signaling that regulates intramembranous ossification, WNT/β-catenin signaling,^49^ in *Dhcr7*^*−/−*^ and *Insig1/2* cKO mice. Recent studies indicate that primary cilia negatively regulate WNT/β-catenin signaling.^37,50,51^ To investigate the activity of WNT/β-catenin signaling pathway, we conducted quantitative RT-PCR for *Axin2*, a readout of WNT/β-catenin signaling activity, and found that its expression was upregulated and downregulated in *Dhcr7*^*−/−*^ and *Insig1/2* cKO osteoblasts, respectively (Fig. 6a, b and Supplementary Fig. S8). To validate the relationship between WNT/β-catenin signaling and cholesterol metabolism, we treated primary osteoblasts from *Dhcr7*^*−/−*^ and *Insig1/2* cKO mice with either WNT3A or lithium chloride (LiCl), a known WNT/β-catenin signaling activator. While both WNT3A and LiCl induced *Axin2* expression five-fold in wild-type controls, *Axin2* expression was upregulated and downregulated in *Dhcr7*^*−/−*^ and *Insig1/2* cKO osteoblasts, respectively (Fig. 6c, d). We confirmed that in mice with *Topgal* reporter for the WNT/β-catenin signaling activity, its activity was increased at osteogenic front of the calvaria in *Dhcr7*^*−/−*^ mice compared to control mice (Fig. 6e). Next, to examine whether *Col1a1* expression was regulated through WNT/β-catenin signaling, we conducted promoter analyses for β-catenin binding on the *Col1a1* promoter and found four putative β-catenin binding sites (Supplementary Fig. S7b). To evaluate these predicted binding sites, we conducted ChIP assays for β-catenin binding to the *Col1a1* promoter region, and found that β-catenin bound at binding sites 1 and 4, and the binding was correlated with the activity of WNT/β-catenin signaling in osteoblasts from *Dhcr7*^*−/−*^ and *Insig1/2* cKO mice (Supplementary Fig. S9). Lastly, to test the functional significance of WNT/β-catenin signaling, we generated and investigated compound mutant mice with a haploinsufficiency of *Axin2* in the *Dhcr7* mutant background and found that normalized WNT/β-catenin signaling restored the accelerated bone formation in *Dhcr7*^*−/−*^ mice (Fig. 6f–h). Altogether, these findings are well supported by the fact that WNT/β-catenin signaling positively regulates osteogenesis.^52^

The bone phenotype in *Dhcr7*- and *Insig1/2*-deficient mice is restored by the normalization of aberrant cholesterol metabolism with simvastatin

**Fig. 7:**
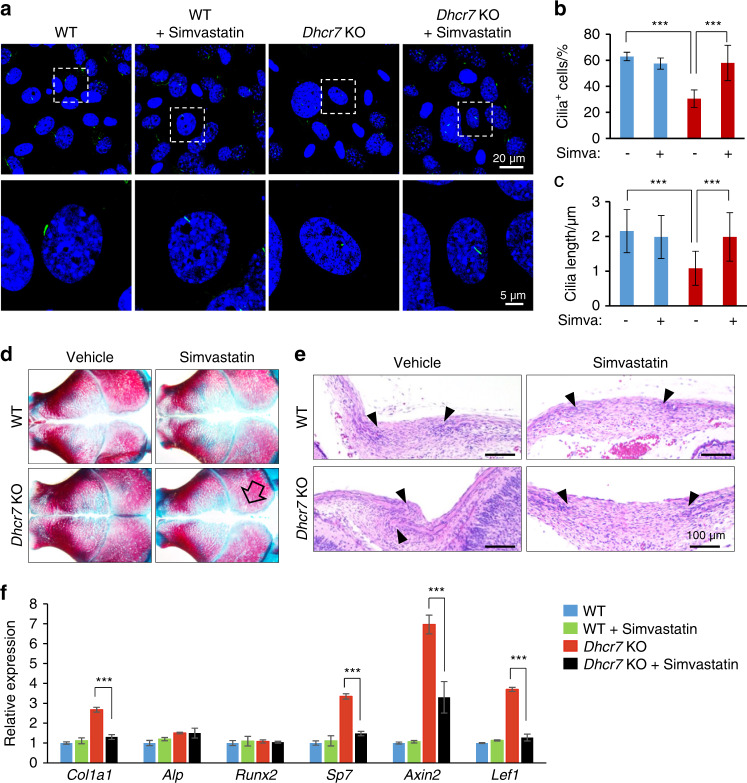
Simvastatin rescues altered bone formation in *Dhcr7* mutant mice. **a** Immunocytochemistry (IC) analyses of primary cilia in osteoblasts from wild-type (WT) control and *Dhcr7* knockout (KO) mice after simvastatin treatment. Primary cilia were stained with anti-acetylated tubulin antibody (green), and nuclei were stained with DAPI (blue). Boxed areas in upper images are enlarged. Scale bars, 20 µm in the upper images and 5 µm in the lower images. **b** Percentage of cells with primary cilia in osteoblasts from WT (blue bars) and *Dhcr7* KO (red bars) mice after treatment with simvastatin. *n* = 124 per group. ***P < 0.001. **c** Quantification of the length of primary cilia in osteoblasts from WT (blue bars) and *Dhcr7* (red bars) KO mice after simvastatin treatment. *n* = 124 per group. ***P < 0.001. **d** Skull staining after simvastatin treatment (10 mg·kg^−1^ body weight, intraperitoneal injection to a pregnant mouse, E12.5‒E18.5). The open arrow indicates rescued calvarial abnormalities. **e** Hematoxylin and Eosin staining of the sagittal sutures of newborn WT and *Dhcr7* KO mice after simvastatin treatment. Arrowheads indicate the osteogenic front. Accelerated bone formation of the sutures (frontal, coronal, and sagittal sutures) was normalized in newborn *Dhcr7* KO mice (*n* = 6/6). Scale bars, 100 µm. **f** Quantitative RT-PCR o**f** the indicated genes in newborn WT (blue bars), WT treated with simvastatin (green bars), *Dhcr7* KO (red bars), and *Dhcr7* KO treated with simvastatin (black bars) mice. *n* = 6 per group. ***P < 0.001.

**Fig. 8:**
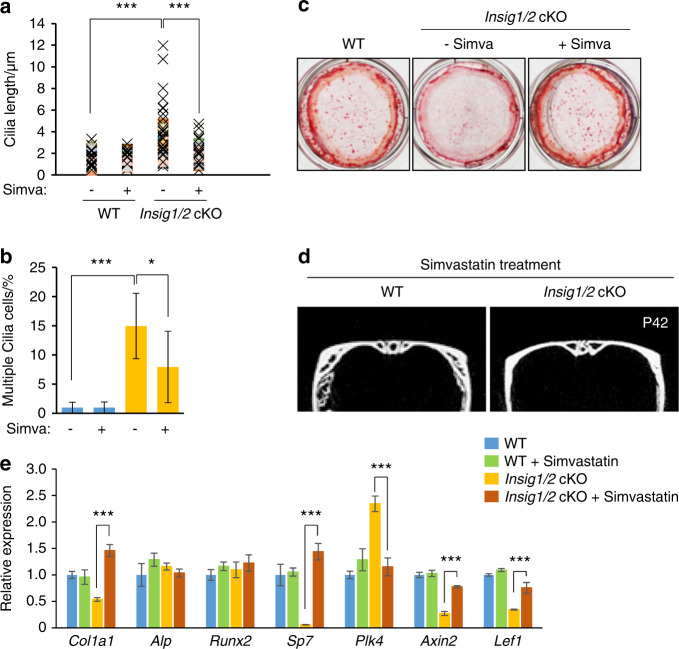
Simvastatin rescues altered bone formation in *Insig1/2* mutant mice. **a** Quantification of ciliary length in WT and *Insig1/2* cKO osteoblasts with/without simvastatin treatment (Simva). *n* = 124 per group. ***P < 0.001. **b** Quantification of cells with multiple cilia with/without simvastatin treatment (Simva) in WT (blue bars) and *Insig1/2* cKO (yellow bars). *n* = 124 per group. *P < 0.05; ***p < 0.001. **c** Alizarin Red staining of osteoblasts isolated from newborn WT and *Insig1/2* cKO frontal bones after induction of osteogenic differentiation with/without simvastatin treatment at Day 28. **d** MicroCT images from WT and *Insig1/2* cKO mice after simvastatin treatment (10 mg·kg^−1^ body weight from P7 to P42). **e** Quantitative RT-PCR of the indicated genes in P42 WT (blue bars), WT treated with simvastatin (green bars), *Insig1/2* cKO (yellow bars), and *Insig1/2* cKO treated with simvastatin (brown bars) mice. *n* = 6 per group. ***P < 0.001.

**Fig. 9:**
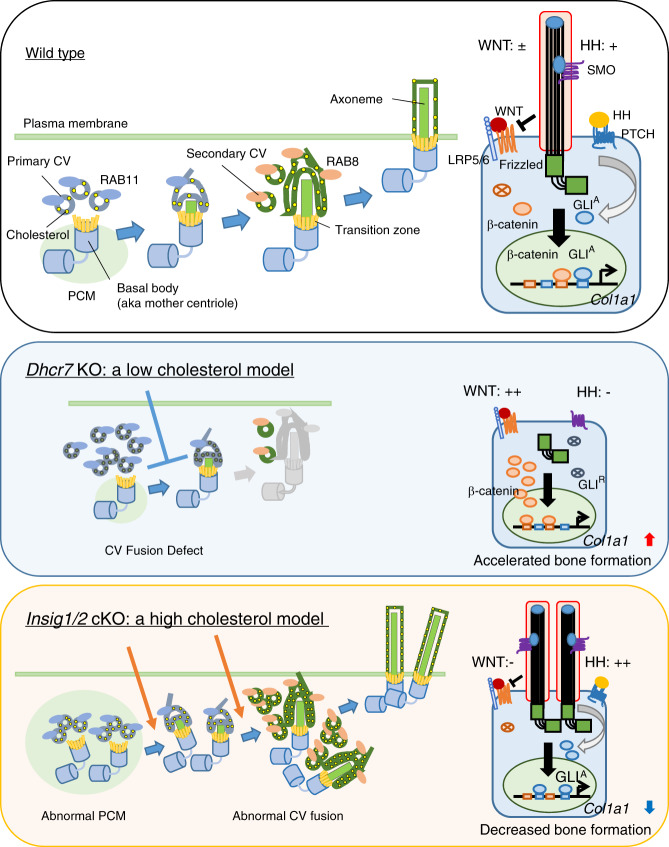
Model of primary cilium formation altered in *Dhcr7*^*−/−*^ and *Insig1/2* mutant osteoblasts. Ciliogenesis starts with the interaction of the basal body (aka mother centriole) with primary ciliary vesicles (CVs), which can be labeled with RAB11, and then the axoneme grows within the ciliary membrane while fusing with secondary CVs, which can be labeled with RAB8. Eventually the elongated primary cilium fuses with the plasma membrane, allowing the distal part of the cilium to interact with the extracellular milieu. In *Dhcr7* knockout (KO) osteoblasts, primary cilia were fewer and shorter than in controls. By contrast, *Insig1/2* conditional KO (cKO) osteoblasts showed supernumerary and longer primary cilia compared to controls. Primary CVs labeled with RAB11 accumulated in the cells, and secondary CVs labeled with RAB8 failed to gather at cilium formation sites in *Dhcr7* KO osteoblasts. Secondary CVs stained with RAB8 accumulated in *Insig1/2* cKO osteoblasts, and the number of basal bodies was abnormally increased in *Insig1/2* cKO osteoblasts. During osteogenesis, WT osteoblasts have a single primary cilium that suppresses WNT/β-catenin signaling and activates HH signaling, which induces *Col1a1* expression. In *Dhcr7* KO osteoblasts, WNT/β-catenin signaling is hyper-activated (WNT: + + ) and HH signaling is compromised (HH: –), while in *Insig1/2* cKO osteoblasts WNT/β-catenin signaling is inhibited (WNT: −) and HH signaling is hyper-activated (HH: + + ).

To test whether abnormalities in primary cilium and bone formation can be restored by the normalization of aberrant cholesterol metabolism, we treated *Dhcr7*^*−/−*^ and *Insig1/2* cKO osteoblasts with simvastatin, which can normalize cholesterol metabolic aberrations by inhibiting the activity of the HMG-CoA reductase. Simvastatin treatment restored primary cilium formation in cultured osteoblasts from *Dhcr7*^*−/−*^ mice (Fig. 7a–c). In addition, the in vivo administration of simvastatin to *Dhcr7*^*−/−*^ mice (10 mg·kg^−1^ body weight per day, intraperitoneal injection to a pregnant mouse, E12.5-E18.5) could normalize the accelerated bone formation in newborn mice (Fig. 7d, e). We confirmed that expression of genes related to bone formation and WNT signaling was restored in *Dhcr7*^*−/−*^ mice treated with simvastatin (Fig. 7f). These results indicate that cholesterol intermediates caused the bone defects in *Dhcr7*^*−/−*^ mice. In *Insig1/2* cKO mice, simvastatin treatment restored both the increased number and length of primary cilia (Fig. 8a, b) and the reduced bone formation in *Insig1/2* cKO osteoblasts (Fig. 8c). Importantly, simvastatin treatment (10 mg·kg^−1^ body weight per day from P7 to P42) improved the decreased bone formation in these mice (Fig. 8d). As expected, the expression of genes related to osteogenesis, cilia and WNT signaling was normalized in *Insig1/2* cKO mice treated with simvastatin (Fig. 8e). Taken together, our results indicate that proper cholesterol metabolic status is crucial for normal primary cilium formation, which is responsible for osteogenesis in osteoblasts (Fig. 9).

## Discussion

Previous studies show that cholesterol metabolism is important for the formation and homeostasis of bone and cartilage during endochondral ossification.^53,54^ For example, mice with a deletion of *Scap*, a regulator for cholesterol biosynthesis, in either postcranial somatic lateral plate mesodermal cells (*Scap*^*F/F*^*;Prx1-Cre* mice) or chondrocytes (*Scap*^*F/F*^*;Col2a1-Cre* mice), display compromised chondrogenesis and disorganized growth plates, resulting in short limbs.^54^ By contrast, increased intracellular cholesterol in mice deficient for *Insig1/2* in postcranial somatic lateral plate mesodermal cells (*Insig1*^*F/F*^*;Insig2*^*−/−*^*;Prx1-Cre* mice) or chondrocytes (*Insig1*^*F/F*^*;Insig2*^*−/−*^*;Col2a1-Cre* mice) show defects in growth plate organization and chondrogenesis, also resulting in short limbs.^54^ Thus, either too much and too low of cholesterol results in short limbs through defective endochondral ossification. In this study, we aimed to study the role of cholesterol in intramembranous ossification using both gain-of-function (*Insig1/2* cKO mice) and loss-of-function (*Dhcr7*^*−/−*^ mice) mouse models, and found that cholesterol metabolism plays a role in intramembranous ossification in a dose-dependent manner. These findings suggest that effects of abnormal cholesterol metabolism differ between intramembranous and endochondral ossification.

7-DHC is a cholesterol intermediate as well as a precursor of vitamin D; 7-DHC is converted to pre-vitamin D_3_ by ultraviolet light on the skin and further converted to active vitamin D_3_ in the liver and kidney.^55^ Previous studies indicate that suppression of DHCR7 activity results in upregulated vitamin D_3_ levels in vivo after birth.^56–58^ While patients with SLOS show a high level of vitamin D_3_ compared with healthy individuals, calcium homeostasis is not affected.^59^ As vitamin D_3_ biosynthesis is inactive during embryogenesis due to lack of exposure to ultraviolet light, and because only maternal 25-hydroxyvitamin D is a source of vitamin D,^60,61^ the effect of vitamin D_3_ on bone development would be minimal. By contrast, while the maternal cholesterol supply partially restores the exogenous cholesterol supply in embryos, embryonic tissues still need to synthesize cholesterol through endogenous cholesterol synthesis pathways in each organ to compensate for the gap between the external supply and their needs.^62–64^ This could be a possible reason why cholesterol metabolic anomalies cause different degree/severity of malformations in different tissues in mice and humans.

Primary cilia transduce extracellular cues as a mechanical sensor, as well as a chemical sensor, for morphogens and growth factors.^65,66^ Therefore, both endochondral and intramembranous ossification are affected in mice with defective primary cilia.^38^ For example, mice deficient for *Kif3a*, a ciliary protein, in osteoblasts display decreased bone formation and osteopenia.^67^ A deficiency for cilia-related protein SPEF2 results in osteoblast differentiation defects.^68^ In this study, we found that HH and WNT/β-catenin signaling is inverted in conditions of the primary cilia in *Dhcr7*^*−/−*^ and *Insig1/2* cKO mice during intramembranous ossification. While both WNT/β-catenin and HH signaling pathways are essential for endochondral ossification as well as for the differentiation and maturation of cultured osteoblasts,^36^ previous mouse genetic studies suggest that WNT/β-catenin signaling may be more dominant than HH signaling for osteogenesis in intramembranous ossification during skull formation.

In regard to HH signaling, previous studies show that HH signaling is compromised in *Dhcr7* mutant mammalian models; for example, HH signaling is inhibited in *Dhcr7*^*−/−*^ mouse embryonic fibroblasts (MEFs)^69^, as well as in mice deficient for *Dhcr7* (*Dhcr7*^*ΔEx8/ΔEx8*^ mice).^70,71^ Our results show that the number of ciliated cells, as well as the length of cilia, is decreased in *Dhcr7*^*−/−*^ osteoblasts, which is responsible for diminished HH signaling. Previous studies indicate that HH signaling is not dominant in regulating intramembranous ossification; for example, mice with loss of *Ihh* (*Ihh*^*−/−*^ mice), which is expressed at the osteogenic front in cranial bones,^72,73^ develop small but normal calvaria,^47,48^ and osteogenesis is not affected during intramembranous ossification.^74^ Mice deficient for *Smo* (*Smo*^*n/c*^*;Wnt1-Cre* mice) display small but normal skulls in contrast to other severe craniofacial defects.^75^ In addition, mice deficient for *Shh* (*Shh*^*n/c*^*;K14-Cre* mice and *Shh*^*F/F*^*;K14-Cre* mice), which is specifically expressed in the epithelium in craniofacial regions, show normal skull formation while they exhibit cleft palate.^2,76,77^ Thus, loss-of-function of HH signaling does not affect calvaria formation. By contrast, gain-of-function of HH signaling results in defective bone formation; for example, mice with an ectopic HH signaling activation in CNC cells (*Smo*^*+/M2*^*;Osr2-IrsCre*) show cleft palate and osteogenesis defects such as the absence of the palatine processes of the premaxilla and maxilla and a short mandible with ossification defects.^46^ In addition, overexpression of *Shh* in the epithelium (*K14-Shh* transgenic mice) results in craniofacial bone defects, cleft palate, short limbs, and polysyndactyly through the increased and expanded SHH signaling activation in the mesenchyme.^78^ Taken together, although HH signaling may contributes to intramembranous ossification at some degree, upregulation and downregulation of HH signaling in *Dhcr7*^*−/−*^ and *Insig1/2* cKO mice is not in agreement with the hypothesis that HH is a factor responsible for cilia-mediated osteogenesis during intramembranous ossification.

As to WNT/β-catenin signaling, previous studies show that it positively regulates osteogenesis; for example, constitutive active WNT/β-catenin signaling in bones results in increased osteogenesis in mice,^79–81^ and WNT/β-catenin signaling inhibition results in a failure of osteogenesis; for example, mice with inactivated β-catenin in mesoderm-derived osteoblast and chondrocyte progenitor cells (*Catnby*^*c/c*^*;Dermo1-Cre* mice) show drastically diminished osteogenesis and ectopic cartilage formation in both intramembranous and endochondral ossification.^82^ *Kif3a*^*−/−*^ and *Kif3a*^*F/F*^*;Wnt1-Cre* mice as well as MEFs or embryonic stem (ES) cells from *Kif3a*^*−/−*^, *Ofd1*^*−/−*^ and *Ift88*^*orpk/orpk*^ mice with no functional primary cilia show hyper-activated WNT/β-catenin signaling, compared to ciliated control cells,^83,84^ as seen in *Dhcr7*^*−/−*^ osteoblasts. Thus, WNT/β-catenin signaling is well correlated with the osteogenic phenotype in *Dhcr7*^*−/−*^ and *Insig1/2* cKO mice during intramembranous ossification.

In summary, we demonstrated the biological significance of intracellular cholesterol metabolism in the regulation of osteogenesis and ciliogenesis using *Dhcr7*^*−/−*^ and *Insig1/2* cKO mice and derived cultured osteoblasts. *Dhcr7*^*−/−*^ osteoblasts displayed fewer and shorter primary cilia compared to controls, while *Insig1/2* cKO osteoblasts exhibited supernumerary and longer primary cilia compared to controls. Our work places a new focus on primary cilium formation regulated by cholesterol metabolism in the bone. The principles learned from this study promise to be fertile ground for future molecular genetic studies of craniofacial bone development, and may lead to the development of innovative preventive and therapeutic approaches for bone diseases and ciliopathies related to cholesterol metabolic aberrations.

## Materials and methods

### Animals

*Dhcr7*^*−/−*^ mice^85^ were a gift from Dr. Forbes D. Porter (The *Eunice Kennedy Shriver* National Institute of Child Health and Human Development, National Institutes of Health, Bethesda, Maryland, USA). *Insig1*^*F/F*^*;Insig2*^*−/−*^ (The Jackson Laboratory, #005939)^12^ and *Wnt1-Cre2* (The Jackson Laboratory, #022501)^22^ mice were obtained from The Jackson Laboratory and crossed to generate *Insig1/2* cKO mice. *Gli1-LacZ* mice (The Jackson Laboratory, #008211)^39^ were obtained from The Jackson Laboratory and crossed with *Dhcr7*^*+/*−^ and *Wnt1-Cre2;Insig1*^*F/+*^*;Insig2*^*−/−*^ mice in order to generate *Dhcr7*^*−/−*^*;Gli1-LacZ*, *Dhcr7*^*+/+*^*;Gli1-LacZ*, *Insig1/2 cKO;Gli1-LacZ*, and *Insig1*^*F/F*^*;Insig2*^*−/−*^*;Gli1-LacZ* mice. *Topgal* (The Jackson Laboratory, #004623)^86^ and *Axin2*^*LacZ/+*^ (The Jackson Laboratory, #009120)^87^ mice were obtained from The Jackson Laboratory and crossed with *Dhcr7*^*−/−*^ mouse line to generate *Dhcr7*^*−/−*^*;Topgal* and *Dhcr7*^*+/+*^*;Topgal*, *Dhcr7*^*−/−*^*;Axin2*^*LacZ/+*^, and *Dhcr7*^*+/+*^*;Axin2*^*LacZ/+*^ mice. Genotyping was performed using PCR primers, as previously described.^12,22,85^ Pregnant females were treated with simvastatin (S6196; Sigma-Aldrich) at a dose of 10 mg/kg^−1^ body weight (BW) from E12.5 to E18.5, or from day 7 to day 42, administered by intraperitoneal injection.

### Skeletal staining

The three-dimensional architecture of the skeleton was examined by modified whole-mount Alcian blue-Alizarin Red S staining, as previously described.^88^

### MicroCT

MicroCT analysis was performed using a Scanco µCT40 unit in the microCT core facility at Baylor College of Medicine. The data were collected at a resolution of 20 µm. Three-dimensional reconstruction was performed using the BoneJ software.

### Histology

H&E, BrdU staining, von Kossa staining, LacZ staining, immunohistochemistry, and the TUNEL assay were performed as previously described.^88,89^ Antibodies used for immunohistochemistry were the anti-COL1A1 rabbit polyclonal (Abcam), anti-RUNX2 rabbit monoclonal (Cell Signaling Technology), anti-SP7 rabbit polyclonal (Abcam), anti-BrdU rat monoclonal (Abcam), and anti-Ki67 rabbit monoclonal (Abcam) antibodies (Supplementary Table S1). Click-iT® Plus TUNEL Assay with Alexa 594 (C10618, Molecular probes) was used to detect apoptotic cells, according to the manufacturer’s instructions. Fluorescence images were obtained using a confocal microscope (Ti-C2, Nikon).

### Cell culture

Primary osteoblasts were obtained from newborn frontal bones. Briefly, the frontal bones were dissected at birth and incubated with 2 mg·mL^−1^ collagenase II solution (Gibco) while shaking at 350 r·min^−1^ at 37 °C for 1 h. Cells were resuspended in minimum essential medium alpha (MEM-α) supplemented with 10% fetal bovine serum (FBS), penicillin/streptomycin, and l-glutamine. For osteogenic differentiation, osteoblasts were cultured in 12-well plates, and osteogenic differentiation was induced with osteogenic induction medium (MEM-α supplemented with 100 µg·mL^−1^ ascorbic acid, 5 mmol·L^−1^ beta-glycerol phosphate, 10% FBS, penicillin/streptomycin, and L-glutamine) for 28 days. To evaluate osteogenic differentiation, alkaline phosphatase staining, Alizarin Red staining, and von Kossa staining were performed. To induce ciliogenesis, osteoblasts were starved of serum for 24 h, followed by immunofluorescent staining or collection of RNA and protein. To evaluate WNT signaling activity, osteoblasts were starved of serum for 24 h and then treated with either 20 mmol·L^−1^ lithium chloride (LiCl) or 20 mmol·L^−1^ NaCl (a negative control) in a serum-free medium for 24 h. For WNT3A treatment, WNT3A-conditioned medium and control medium were prepared from murine WNT3A-overexpressing cells (L-Wnt3a cells: the American Type Culture Collection [ATCC]) and from control L-cells (ATCC), according to manufacturer’s instructions, and used at 10% for each assay for 24 h, respectively. For the cell proliferation assay, osteoblasts were plated onto 96-well plates at a density of 5 000 cells per well and then counted by CCK8 (Dojindo Molecular Technologies) at 24, 48, and 72 h. For the BrdU incorporation assay, osteoblasts were plated onto a 35-mm culture dish at a density of 10 000 cells per dish for 2 days, and then 100 µg·mL^−1^ BrdU was added for 1 h. To generate *Insig1/2* cKO osteoblasts from *Insig1*^*F/F*^*;Insig2*^*−/−*^ osteoblasts, the *Ad5-CMV-LacZ-Cre* virus (Vector Development Laboratory) was transduced at 300 multiplicity of infection (MOI) for 2 days. The Cre recombinase efficiency was evaluated with LacZ staining.

### Organ cultures

Calvarial explants were dissected out from newborn *Dhcr7*^*−/−*^ pups and incubated in BGjB medium (Gibco) supplemented with 10% FBS, 0.1 mg·mL^−1^ ascorbic acid, and penicillin/streptomycin, in a rotor incubator at 37 °C for 3 days.

### Immunocytochemical analysis

The ciliated osteoblasts plated onto ibiTreat 4-well μ-slides (ibidi) were obtained through 24-h serum starvation. The antibodies used in immunocytochemistry were the anti-γ-tubulin mouse monoclonal (Sigma-Aldrich), anti-acetylated tubulin mouse monoclonal (Sigma-Aldrich), anti-RAB8 rabbit monoclonal (Cell signaling Technology), anti-RUNX2 rabbit monoclonal (Cell Signaling Technology), and anti-RAB11 rabbit polyclonal (Abcam) antibodies (Supplementary Table S1). Nuclei were counterstained with DAPI. The number and length of primary cilia were measured by NIS-Elements (Nikon) under a confocal microscope (Ti-C2, Nikon).

### Quantitative RT-PCR

Total messenger RNA was prepared as previously described.^88^ Quantitative RT-PCR was conducted using the following primers: *Runx2*, 5ʹ-CGGACGAGGCAAGAGTTTCA-3ʹ and 5ʹ-GGATGAGGAATGCGCCCTAA-3ʹ; *Alp*, 5ʹ-CTGAAGGCTCTCTTCACTCCAA-3ʹ and 5ʹ-AGGCGACAGGTGAAGAAACA-3ʹ; *Col1a1*, 5ʹ-GAAGATGTAGGAGTCGAGGGAC-3ʹ and 5ʹ-CCTTGGAAACCTTGTGGACC-3ʹ; *Col1a2*, 5ʹ-CAAAGGCGTGAAAGGACACAG-3ʹ and 5ʹ-GCCAGTGAGCCCATTTGTTC-3ʹ; *Bglap*, 5ʹ-CCTAGCAGACACCATGAGGAC-3ʹ and 5ʹ-GTTTGGCTTTAGGGCAGCAC-3ʹ; *Sparc*, 5ʹ-GCCTACCACAAGGCAAGGAA-3ʹ and 5ʹ-CAGGTACCCCTGTCTCCTCC-3ʹ; *Sp7*, 5ʹ-GCCTGACTCCTTGGGACC-3ʹ and 5ʹ-TAGTGAGCTTCTTCCTCAAGCA-3ʹ; *Spp1*, 5ʹ-AGTGACTGATTCTGGCAGCTC-3ʹ and 5ʹ-ATTGCTTGGAAGAGTTTCTTGCT-3ʹ; *Plk1*, 5ʹ- CCT TTG AGA CCT CGT GCC TA-3ʹ and 5ʹ- GGT TCT CCA CAC CTT TAT TGA GGA-3ʹ; *Plk4*, 5ʹ-AGACCGGCGGGAATTTTTCA-3ʹ and 5ʹ-TAAAGTCCTCGATCCTCTCCCC-3ʹ; *Sass6*, 5ʹ-GGAGAGGAGAGGGAGCGTTA-3ʹ and 5ʹ-CCTTGGAGTCTCTTTCGCGT-3ʹ; *Stil*, 5ʹ-TGCCTACGAGCCCAAATCAC-3ʹ and 5ʹ-TAGGCTTCACAGGCACACAC-3ʹ; *Axin2*, 5ʹ-GACGGACAGTAGCGTAGATGG-3ʹ and 5ʹ-CAGACTATGGCGGCTTTCCA-3ʹ; *Lef1*, 5ʹ-CGGGAAGAGCAGGCCAAATA-3ʹ and 5ʹ-CTGGGACCTGTACCTGAAGTC-3ʹ; *Gli1*, 5ʹ-CACTGAGGACTTGTCCAGCTTG-3ʹ and 5ʹ-AGCTGGGCAGTTTGAGACC-3ʹ; *Ptch1*, 5ʹ-TAGCCCTGTGGTTCTTGTCC-3ʹ and 5ʹ-TGTGGTCATCCTGATTGCAT-3ʹ; *Wnt1*, 5ʹ-ACTCATTGTCTGTGGCCCTG-3ʹ and 5ʹ-TATGTTCACGATGCCCCACC-3ʹ; *Wnt3a*, 5ʹ-GATCTGGTGGTCCTTGGCTG-3ʹ and 5ʹ- ACCCATCTATGCCATGCGAG-3ʹ; *Wnt7b*, 5ʹ-CACACTCTGGTCAACCTCCC-3ʹ and 5ʹ- CAGCCTCTCGACTCCCTACT-3ʹ; *Wnt10b*, 5ʹ-TCTGGATCACTCCCTCCCTTT-3ʹ and 5ʹ- GTTACCACCTGGCGTCCC-3ʹ; *Wnt16*, 5ʹ-TATGAGCTGAGTAGCGGCAC-3ʹ and 5ʹ- TCCAGCAGGTTTTCACAGCA-3ʹ; *Fzd3*, 5ʹ-GCAGATAGGTGGGCACAGTT-3ʹ and 5ʹ- ATAGGGTGGAAGGGCTCCAT-3ʹ; *Fzd7*, 5ʹ-GGGGCGAGAGATGGTTTTGA-3ʹ and 5ʹ-AGGCTACAGACAGAGCGGTA-3ʹ; *Fzd9*, 5ʹ-TCACCGTGTTCACCTTCCTG-3ʹ and 5ʹ- GCTTCTCCGTATTGGTGCCT-3ʹ and *Gapdh*, 5ʹ-AACTTTGGCATTGTGGAAGG-3ʹ and 5ʹ-ACACATTGGGGGTAGGAACA-3ʹ.

### Immunoblotting

Immunoblots were obtained as previously described.^89^ The antibodies used for immunoblotting were as follows: anti-collagen type I rabbit polyclonal (Abcam), anti-INSIG1 rabbit polyclonal (Abcam), anti-INSIG2 rabbit polyclonal (Abcam), anti-DHCR7 rabbit polyclonal (Abcam), anti-GLI1 rabbit polyclonal (Abcam), anti-non-phosphorylated (active) beta-catenin rabbit polyclonal (Cell Signaling Technology), anti-CREB rabbit polyclonal (Cell Signaling Technology), anti-SaK (aka PLK4) mouse monoclonal (Santa Cruz Biotechnology), and anti-GAPDH mouse monoclonal (Millipore) (Supplementary Table S1). Cell fractionation was performed using a NE-PER^TM^ nuclear and cytoplasmic extraction kit (Thermo Scientific).

### Promoter analysis

The UCSC genome browser was used to obtain the genomic sequences of the *Col1a1* murine gene (NC_000077.6) and the *Plk4* murine gene (NC_000069.6), including the 5-kbp sequences upstream of the respective transcription start sites. The sequences were then mapped to seven additional mammalian genomes [human (Build 38), chimpanzee (Build 2.1.4), orangutan (Build 2.0.2), rhesus macaque (Build 1.0), rat (Build 5), dog (Build 3.1), and horse (Build equCab2)] with the BLAST tool, as previously described.^90^ Multiple alignments for these sequences were obtained using the Clustal Omega tool with default parameters and settings. LEF1 binding motifs (minimal core sites: 5ʹ-CAAAG-3ʹ and 5ʹ-CTTTG-3ʹ; optimal sites: 5ʹ-CTTTGWW-3ʹ and 5ʹ-WWCAAAG-3ʹ, W = A/T), the GLI-binding motif (5ʹ-CACCACCCA-3ʹ),^91,92^ and the SRE consensus sequence (5ʹ-TCACNCCAC-3ʹ)^93,94^ were searched in the aligned DNA sequences, as previously described.^90^

### Chromatin immunoprecipitation assay

At Day 3 of osteogenic differentiation, the osteoblast extracts were incubated with either active β-catenin (Cell signaling technology), GLI1 (Abcam), or normal rabbit IgG as a negative control (Santa Cruz Biotechnology) overnight at 4 °C, followed by precipitation with magnetic beads. The osteoblast extracts were incubated with either mouse SREBP1 and SREBP2 antibodies (Santa Cruz Biotechnology) or normal mouse IgG (Santa Cruz Biotechnology) overnight at 4 °C, followed by precipitation with magnetic beads. Washing and elution of the immune complexes, as well as precipitation of DNA, were performed according to standard procedures, as previously described.^90^ The putative LEF1/β-catenin binding sites in the immune complexes were detected by PCR using the following primers: *Col1a1* site 1, 5ʹ-AGCAGACGGGAGTTTCTCCT-3ʹ and 5ʹ-GCAGCTGACTTCAGGGATGT-3ʹ (–117 bp to + 93 bp); site 2, 5ʹ-CAGGCTTCCTGCAACAAACT-3ʹ and 5ʹ-AGGGGGTGCCTATCTGTTCT-3ʹ (–985 bp to -736 bp); site 3, 5ʹ-GTCCTTCCATTGCTGTCTCC-3ʹ and 5ʹ-CCATCCAAGATTCCATTGCT-3ʹ (–1814 bp to -1569 bp); and site 4, 5ʹ-TGGAGATTCTGGCTTTTGCT-3ʹ and 5ʹ-TGCAGCATGACAGAGAGAGG-3ʹ (–2756 bp to –2517 bp). The putative GLI-binding sites on the *Col1a1* promotor were detected by the following primers: 5ʹ-CGGGACTTTCTCCTCGGGG-3ʹ (–111 bp to –94 bp) and 5ʹ-GGGGTTAGCTTCGGCTCA-3ʹ (–59 bp to –42 bp). The putative SREs on the *Plk4* promotor in the immune complexes were detected by PCR using the following primers: site 1, 5ʹ-AAACCCACTTCCGGCCTAGA-3ʹ (–322 bp to –303 bp) and 5ʹ-TGAAAAATTCCCGCCGGTCT-3ʹ (–210 bp to –191 bp); and site 2, 5ʹ-GCTTGCAGGATAACGTGTTCATT-3ʹ (–1402 bp to –1380 bp) and 5ʹ-AATAAGAGGAATAGGCTAGCGGG-3ʹ (–1275 bp to –1262 bp). The position of the PCR fragments corresponds to NCBI mouse genome Build 38 (mm10).

### Statistics

The two-tailed student’s *t-*test was applied for statistical analysis. A *P*-value < 0.05 was considered statistically significant. For all graphs, data are represented as mean ± standard deviation.

### Study approval

All animal experiments were reviewed and approved by the Animal Welfare Committee and the Institutional Animal Care and Use Committee of UTHealth.

## Supplementary information

Supplemental Text

Supplemental Tables S1 and S2

Supplemental Tables S1 and S2
