# Supplementary material for: Disruption of Dhcr7 and Insig1/2 in cholesterol metabolism causes defects in bone formation and homeostasis through primary cilium formation
Source: Bone Res. 2020 Jan 2;8:1. doi: 10.1038/s41413-019-0078-3 (PMC6946666; doi:10.1038/s41413-019-0078-3)
Supplement: Supplementary file 1 — Supplemental Text [file 41413_2019_78_MOESM1_ESM.docx]

**Disruption of *Dhcr7* and *Insig1/2* in cholesterol metabolism causes defects in bone formation and homeostasis through primary cilium formation**

Akiko Suzuki,^1,2^ Kenichi Ogata,^1,2^ Hiroki Yoshioka,^1,2^ Junbo Shim,^1,2^, Christopher A. Wassif^3^, Forbes D. Porter^3^, and Junichi Iwata^1,2,4,5^

^1^Department of Diagnostic & Biomedical Sciences, ^2^Center for Craniofacial Research, The University of Texas Health Science Center at Houston, School of Dentistry, Houston, Texas, USA; ^3^Division of Translational Medicine, *Eunice Kennedy Shriver* National Institute of Child Health and Human Development, Bethesda, Maryland, USA; ^4^Pediatric Research Center, The University of Texas Health Science Center at Houston, McGovern Medical School, Houston, Texas, USA; ^5^MD Anderson Cancer Center UTHealth Graduate School of Biomedical Sciences, Houston, Texas, USA

**Supplementary Information**

**Fig. S1.** Phenotypic analysis of *Dhcr7^-/-^* mice. **a** Picture of newborn wild-type (WT) control and *Dhcr7^-/-^* knockout (KO) mice. *Dhcr7* KO mice exhibit a suckling defect (no milk in the stomach) and die within 24 hours after birth. **b** Body weights of newborn WT (blue bar) and *Dhcr7* KO (red bar) mice (n=14 per genotype). ***p*<0.01. **c** von Kossa staining of the sagittal sutures of E16.5 WT and *Dhcr7* KO mice. Arrows indicate the osteogenic front. Nuclei were stained with nuclear fast red. Scale bars, 500 µm. **d** Hematoxylin and Eosin staining of frontal bone explants from newborn WT and *Dhcr7* KO mice after 3 days in culture.

**Fig. S2.** No change in cell proliferation and apoptosis in osteoblasts in the frontal bones of *Dhcr7^-/-^* mice. **a** Hematoxylin and Eosin (H&E), Ki67, BrdU, and TUNEL staining in the frontal bones of E14.5 wild-type (WT) control and *Dhcr7* knockout (KO) mice. Nuclei were counterstained with 0.04% methylene blue. Scale bars, 100 µm in H&E, 50 µm in Ki67, BrdU, and TUNEL. **b** Quantification of Ki67-, BrdU-, and TUNEL-positive cells in the frontal bones of E14.5 wild-type (WT) control (blue bars) and *Dhcr7^-/-^* knockout (KO; red bars) mice. More than 20 images were randomly analyzed in three independent experiments. **c** H&E, Ki67, BrdU, and TUNEL staining in the frontal bones of E16.5 wild-type (WT) control and *Dhcr7* knockout (KO) mice. Nuclei were counterstained with 0.04% methylene blue. Scale bars, 100 µm in H&E, 50 µm in Ki67, BrdU, and TUNEL. **d** Quantification of Ki67-, BrdU-, and TUNEL-positive cells in the frontal bones of E16.5 wild-type (WT) control (blue bars) and *Dhcr7^-/-^* knockout (KO; red bars) mice. More than 20 images were randomly analyzed in three independent experiments.

**Fig. S3.** Accelerated osteogenic differentiation in *Dhcr7^-/-^* osteoblasts. **a** Cell proliferation assays in cultured osteoblasts from WT (blue line) and *Dhcr7* KO (red line) calvaria. n=6 per group. **b** BrdU incorporation assays in cultured osteoblasts from WT (blue bar) and *Dhcr7* KO (red bar) calvaria. NS, not significant. n=6 per group. **c**, **d** qRT-PCR for osteogenic genes in WT (blue bars) and *Dhcr7* KO (red bars) calvaria at E15.5 (c) and P0 (d). n=6 per group. **p*<0.05; ****p*<0.001. **e** von Kossa staining of osteoblasts isolated from newborn WT and *Dhcr7* KO calvaria after induction of osteogenic differentiation at Day 0, 7, and 14.

**Fig.S4.** No change in cell proliferation and apoptosis in *Insig1/2* KO mice. **a** Hematoxylin and Eosin (H&E), Ki67, BrdU, and TUNEL staining in the frontal bones of E14.5 wild-type (WT) control and *Insig1/2* conditional knockout (cKO) mice. Nuclei were counterstained with 0.04% methylene blue. Scale bars, 100 µm in H&E, 50 µm in Ki67, BrdU, and TUNEL. **b** Quantification of Ki67-, BrdU-, and TUNEL-positive cells in (A) at E14.5. WT; blue bars, cKO; yellow bars. More than 20 images were randomly analyzed in three independent experiments. **c** Hematoxylin and Eosin (H&E), Ki67, BrdU, and TUNEL staining in the frontal bones of E16.5 wild-type (WT) control and *Insig1/2* conditional knockout (cKO) mice. Nuclei were counterstained with 0.04% methylene blue. Scale bars, 100 µm in H&E, 50 µm in Ki67, BrdU, and TUNEL. **d** Quantification of Ki67-, BrdU-, and TUNEL-positive cells in (C) at E16.5. WT; blue bars, cKO; yellow bars. More than 20 images were randomly analyzed in three independent experiments. **e** Cell proliferation assays in cultured osteoblasts from WT (blue line) and *Insig1/2* cKO (yellow line) mice. n=6 per group. **f** BrdU incorporation assays in cultured osteoblasts from WT and *Insig1/2* cKO mice. Graph shows quantification of BrdU staining. More than 20 areas were randomly analyzed in three independent experiments. Nuclei were stained with hematoxylin. WT; blue bars, cKO; yellow bars. Scale bars, 50 µm.

**Fig. S5.** Phenotypic analysis of *Insig1/2* mutant osteoblasts with an adenovirus-Cre system. **a** Cell proliferation assays at Day 0–3 after adenovirus Cre (yellow line) or control (blue line) administration. n=6 per group. **b** BrdU incorporation assay after adenovirus Cre or control administration. Scale bars, 100 µm. **c** Quantification of BrdU-positive cells in (B) after adenovirus Cre (yellow bar) or control (blue bar) administration. More than 20 areas were randomly analyzed in three independent experiments. **d** Alizarin Red staining to examine osteogenic differentiation after adenovirus Cre or control administration. Osteoblasts isolated from the *Insig1^F/F^;Insig2^-/-^* frontal bones were cultured with osteogenic differentiation medium for 21 days with/without adenovirus-Cre. β-garactosidase (LacZ) staining was performed to examine Cre recombinase efficiency. **e** Immunocytochemical analyses of primary cilia after adenovirus Cre or control administration. Primary cilia were stained with anti-acetylated tubulin antibody (green), and nuclei were stained with DAPI (blue). Scale bars, 5 µm.

**Fig. S6.** Abnormal increase in number of centrioles in *Insig1/2* mutant osteoblasts. **a** Immunocytochemical analyses for centrioles and chromosomal spindle fibers during mitosis. Centrioles and chromosomal spindle fibers were stained with γ-tubulin (red) and acetylated tubulin (green), respectively. DAPI was used for nuclei staining. Arrows indicate increased centrioles. Scale bars, 5 µm. **b** Quantitative RT-PCR analyses for gene expression of the indicated genes in *Insig1/2* conditional KO (cKO; yellow bar) compared with WT control (blue bars) osteoblasts. n=6 per group. NS, not significant.

**Fig. S7.** Bioinformatics analysis for binding sites of GLI and LEF1 in the *Col1al* promoter region*.* **a**, **b** Promoter sequences of the binding sites of GLI (a) and LEF1 (b) that are conserved in all eight species are highlighted in green.

**Fig. S8.** Elevated WNT expression in osteoblasts of *Dhcr7* KO mice. **a** Immunohistochemical analysis for acetylated tubulin (green) and RUNX2 (red) in the osteogenic front region of E14.5 WT, *Dhcr7* KO and *Insig1/2* cKO mice. Nuclei were counterstained with DAPI (blue). Scale bars, 5 µm. **b.** Quantification of cells with cilia in WT (blue bar), *Dhcr7* KO (red bar), and *Insig1/2* cKO (yellow bar) at E14.5 in the calvaria. n=10 per group. ****p*<0.001. **c.** Quantification of ciliary length in WT (blue bar), *Dhcr7* KO (red bar), and *Insig1/2* cKO (yellow bar) osteoblasts at E14.5 in the calvaria. n=10 per group. ***p*<0.01, ****p*<0.001. **d** Quantitative RT-PCR of the indicated genes in newborn WT (blue bars) and *Dhcr7* KO (red bars) mice. n=6 per group. ****p*<0.001.

**Fig. S9.** WNT/β-catenin signaling is regulated through AHI1 in *Dhcr7^-/-^* and *Insig1/2* mutant osteoblasts. **a** ChIP assays of IgG control and active β-catenin (ABC) for binding sites 1–4 (BS 1–4) of the *Col1a1* promoter region in *Dhcr7^-/-^* knockout (KO; red bars) and wild-type control (WT; blue bars) osteoblasts. n=3 per group. ***p*<0.01; ****p*<0.001. **b** ChIP assays of IgG control and active β-catenin (ABC) for binding sites 1–4 (BS 1–4) of the *Col1a1* promoter region in *Insig1/2* cKO (yellow bars) and WT control (blue bars) osteoblasts. n=3 per group. ***p*<0.01.
