# Supplementary material for: Disruption of Dhcr7 and Insig1/2 in cholesterol metabolism causes defects in bone formation and homeostasis through primary cilium formation
Source: Bone Res. 2020 Jan 2;8:1. doi: 10.1038/s41413-019-0078-3 (PMC6946666; doi:10.1038/s41413-019-0078-3)
Supplement: Supplementary file 2 — Supplemental Tables S1 and S2 [file 41413_2019_78_MOESM2_ESM.docx]

**Supplementary Table S1.** Antibodies used in this study.

| **Antibody name** | **Provider** | **Catalog number** | **Application** | **Concentration** |
| --- | --- | --- | --- | --- |
| Acetylated tubulin | Sigma-Aldrich | T6793 | IF | 1:2000 |
| AHI | Santa Cruz Biotechnology | sc-515382 | WB | 1:500 |
| BrdU | Abcam | ab6321 | IHC | 1:500 or 1:1000 |
| Collagen type I | Abcam | ab21286 | WB | 1:1000 |
|  |  |  | IHC | 1:200 |
| CREB | Cell Signaling Technology | 9197 | WB | 1:1000 |
| DHCR7 | Abcam | ab103296 | WB | 1:500 |
| GAPDH | MilliporeSigma | MAB374 | WB | 1:6000 |
| GLI1 | Abcam | ab49314 | ChIP | 5 μg |
|  |  |  | WB | 1:500 |
| INSIG1 | Abcam | ab70784 | WB | 1:500 |
| INSIG2 | Abcam | ab86415 | WB | 1:500 |
| Ki67 | Abcam | ab16667 | IHC | 1:300 |
| non-phosphorylated active β-catenin | Cell Signaling Technology | 8814 | ChIP | 1:100 |
|  |  |  | WB | 1:1000 |
| RAB8 | Cell Signaling Technology | 6975 | IF | 1:100 |
| RAB11 | Abcam | ab3612 | IF | 1:100 |
| RUNX2 (D1L7F) | Cell Signaling Technology | 12556 | IHC | 1:250 |
| γ-tubulin | Sigma-Aldrich | T5326 | IF | 1:1000 |
| SaK | Santa Cruz Biotechnology | sc-100413 | WB | 1:500 |
| SP7 | Abcam | ab22552 | IHC | 1:100 |
| SREBP1 | Santa Cruz Biotechnology | sc-13551x | ChIP | 5 μg |
| SREBP2 | Santa Cruz Biotechnology | sc-13552x | ChIP | 5 μg |
| Rabbit IgG, biotinylated | Vector Laboratories | BA-1000 | IHC | 1:1000 |
| Rat IgG, biotinylated | Vector Laboratories | BA-9400 | IHC | 1:300 |
| Mouse IgGb2, Alexa Fluor 488 | Thermo Fisher | A-21141 | IF | 1:500 |
| Mouse IgGb1, Alexa Fluor 568 | Thermo Fisher | A-21124 | IF | 1:500 |
| Rabbit IgG (H+L), Alexa Fluor 568 | Thermo Fisher | A-11011 | IF | 1:500 |
| Mouse IgG, HRP | Cell Signaling Technology | 7976 | WB | 1:50000 |
| Rabbit IgG, HRP | Cell Signaling Technology | 7074 | WB | 1:50000 |

**Supplementary Table S2.** Primer pairs used in this study.

| **Gene** | **Forward primer** | **Reverse primer** |
| --- | --- | --- |
| *Alp* | 5’-CTGAAGGCTCTCTTCACTCCAA-3’ | 5’-AGGCGACAGGTGAAGAAACA-3’ |
| *Ahi1* | 5’-TATGATTTCCAGGTAGCCCAGC-3’ | 5’-TTTGCAGCACAGGATCGTATCA-3’ |
| *Axin2* | 5’-GACGGACAGTAGCGTAGATGG-3’ | 5’-CAGACTATGGCGGCTTTCCA-3’ |
| *Bglap* | 5’-CCTAGCAGACACCATGAGGAC-3’ | 5’-GTTTGGCTTTAGGGCAGCAC-3’ |
| *Col1a1* | 5’-GAAGATGTAGGAGTCGAGGGAC-3’ | 5’-CCTTGGAAACCTTGTGGACC-3’ |
| *Col1a2* | 5’-CAAAGGCGTGAAAGGACACAG-3’ | 5’-GCCAGTGAGCCCATTTGTTC-3’ |
| *Fzd3* | 5’-GCAGATAGGTGGGCACAGTT-3’ | 5’- ATAGGGTGGAAGGGCTCCAT-3’ |
| *Fzd7* | 5’-GGGGCGAGAGATGGTTTTGA-3’ | 5’-AGGCTACAGACAGAGCGGTA-3’ |
| *Fzd9* | 5’-TCACCGTGTTCACCTTCCTG-3’ | 5’- GCTTCTCCGTATTGGTGCCT-3’ |
| *Gapdh* | 5’-AACTTTGGCATTGTGGAAGG-3’ | 5’-ACACATTGGGGGTAGGAACA-3’ |
| *Gli1* | 5’-CACTGAGGACTTGTCCAGCTTG-3’ | 5’-AGCTGGGCAGTTTGAGACC-3’ |
| *Lef1* | 5’-CGGGAAGAGCAGGCCAAATA-3’ | 5’-CTGGGACCTGTACCTGAAGTC-3’ |
| *Plk1* | 5’- CCTTTGAGACCTCGTGCCTA-3’ | 5’- GGTTCTCCACACCTTTATTGAGGA-3’ |
| *Plk4* | 5’-AGACCGGCGGGAATTTTTCA-3’ | 5’-TAAAGTCCTCGATCCTCTCCCC-3’ |
| *Ptch1* | 5’-TAGCCCTGTGGTTCTTGTCC-3’ | 5’-TGTGGTCATCCTGATTGCAT-3’ |
| *Runx2* | 5’-CGGACGAGGCAAGAGTTTCA-3’ | 5’-GGATGAGGAATGCGCCCTAA-3’ |
| *Sass6* | 5’-GGAGAGGAGAGGGAGCGTTA-3’ | 5’-CCTTGGAGTCTCTTTCGCGT-3’ |
| *Sp7* | 5’-GCCTGACTCCTTGGGACC-3’ | 5’-TAGTGAGCTTCTTCCTCAAGCA-3’ |
| *Sparc* | 5’-GCCTACCACAAGGCAAGGAA-3 | 5’-CAGGTACCCCTGTCTCCTCC-3’ |
| *Spp1* | 5’-AGTGACTGATTCTGGCAGCTC-3’ | 5’-ATTGCTTGGAAGAGTTTCTTGCT-3’ |
| *Stil* | 5’-TGCCTACGAGCCCAAATCAC-3’ | 5’-TAGGCTTCACAGGCACACAC-3’ |
| *Wnt1* | 5’-ACTCATTGTCTGTGGCCCTG-3’ | 5’-TATGTTCACGATGCCCCACC-3’ |
| *Wnt3a* | 5’-GATCTGGTGGTCCTTGGCTG-3’ | 5’- ACCCATCTATGCCATGCGAG-3’ |
| *Wnt7b* | 5’-CACACTCTGGTCAACCTCCC-3’ | 5’- CAGCCTCTCGACTCCCTACT-3’ |
| *Wnt10b* | 5’-TCTGGATCACTCCCTCCCTTT-3’ | 5’- GTTACCACCTGGCGTCCC-3’ |
| *Wnt16* | 5’-TATGAGCTGAGTAGCGGCAC-3’ | 5’- TCCAGCAGGTTTTCACAGCA-3’ |
