# Supplementary material for: Disruption of Dhcr7 and Insig1/2 in cholesterol metabolism causes defects in bone formation and homeostasis through primary cilium formation
Source: Bone Res. 2020 Jan 2;8:1. doi: 10.1038/s41413-019-0078-3 (PMC6946666; doi:10.1038/s41413-019-0078-3)
Supplement: Supplementary file 3 — Supplemental Tables S1 and S2 [file 41413_2019_78_MOESM3_ESM.pdf]

Figure S1

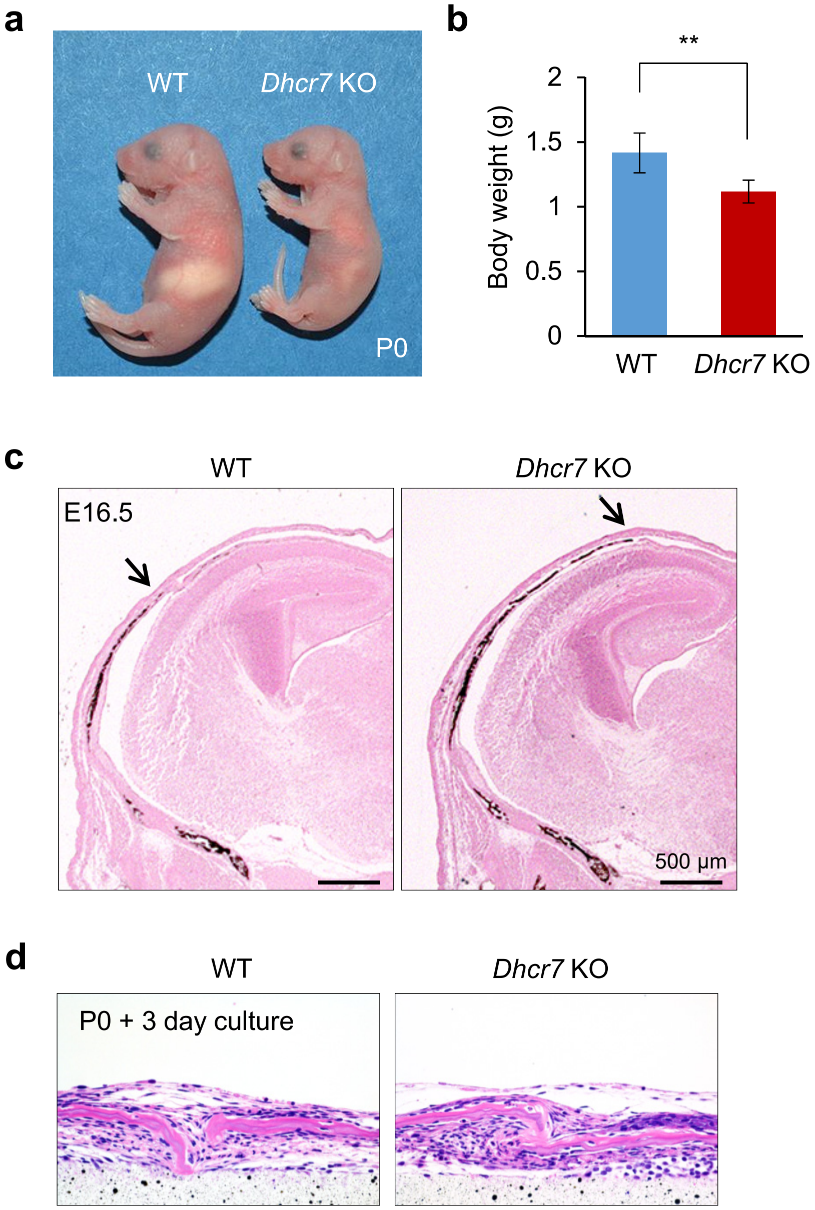
a
WT
Dhcr7 KO
P0
b
**
Body weight (g)
2
1.5
1
0.5
0
WT
Dhcr7 KO
c
WT
Dhcr7 KO
E16.5
500 µm
d
WT
Dhcr7 KO
P0 + 3 day culture

Figure S2

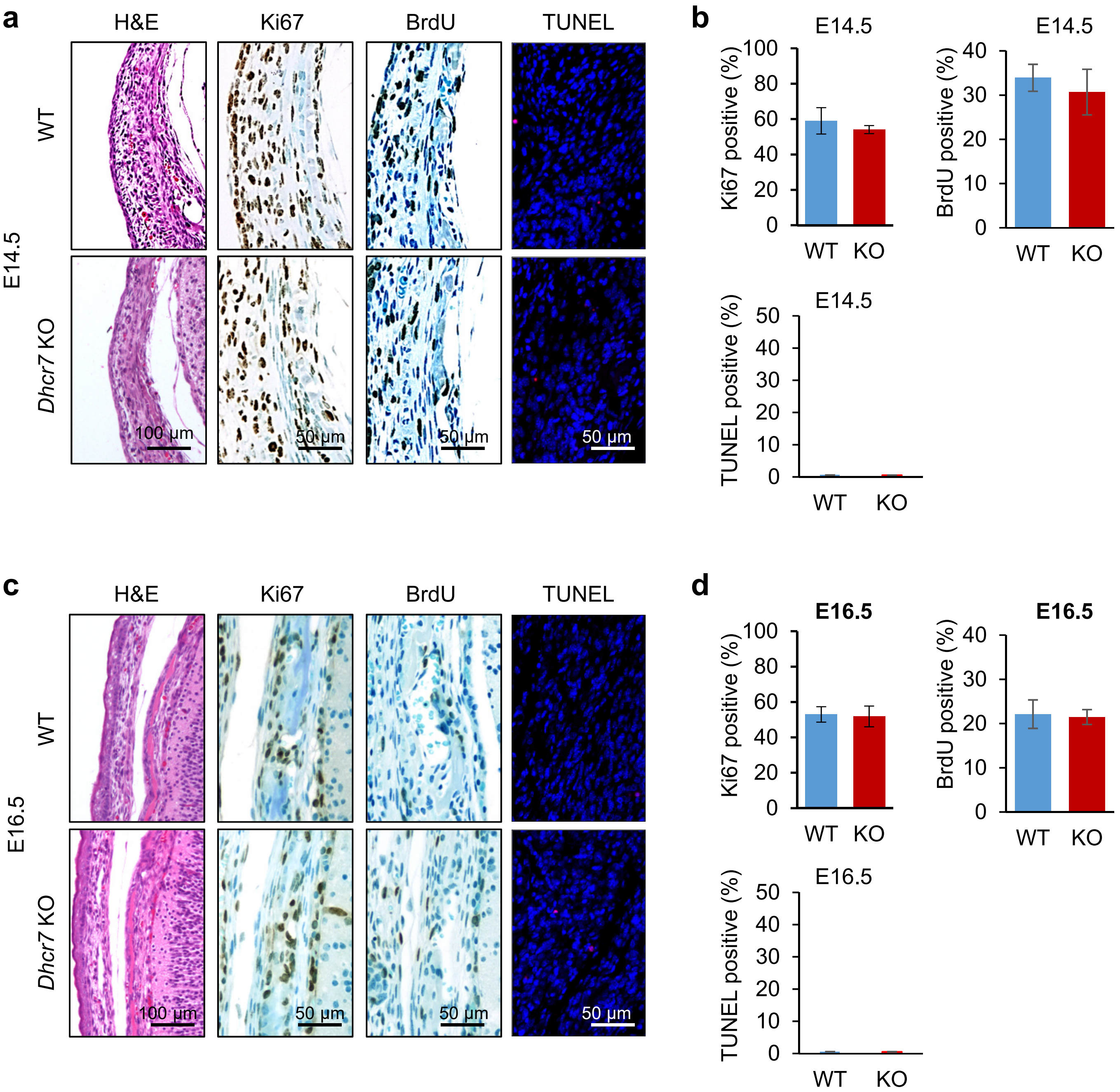
a
H&E
Ki67
BrdU
TUNEL
WT
E14.5
Dhcr7 KO
100 µm
50 µm
50 µm
50 µm
b
E14.5
Ki67 positive (%)
100
80
60
40
20
0
WT
KO
E14.5
BrdU positive (%)
40
30
20
10
0
WT
KO
E14.5
TUNEL positive (%)
50
40
30
20
10
0
WT
KO
c
H&E
Ki67
BrdU
TUNEL
WT
E16.5
Dhcr7 KO
100 µm
50 µm
50 µm
50 µm
d
E16.5
Ki67 positive (%)
100
80
60
40
20
0
WT
KO
E16.5
BrdU positive (%)
40
30
20
10
0
WT
KO
E16.5
TUNEL positive (%)
50
40
30
20
10
0
WT
KO

Figure S3

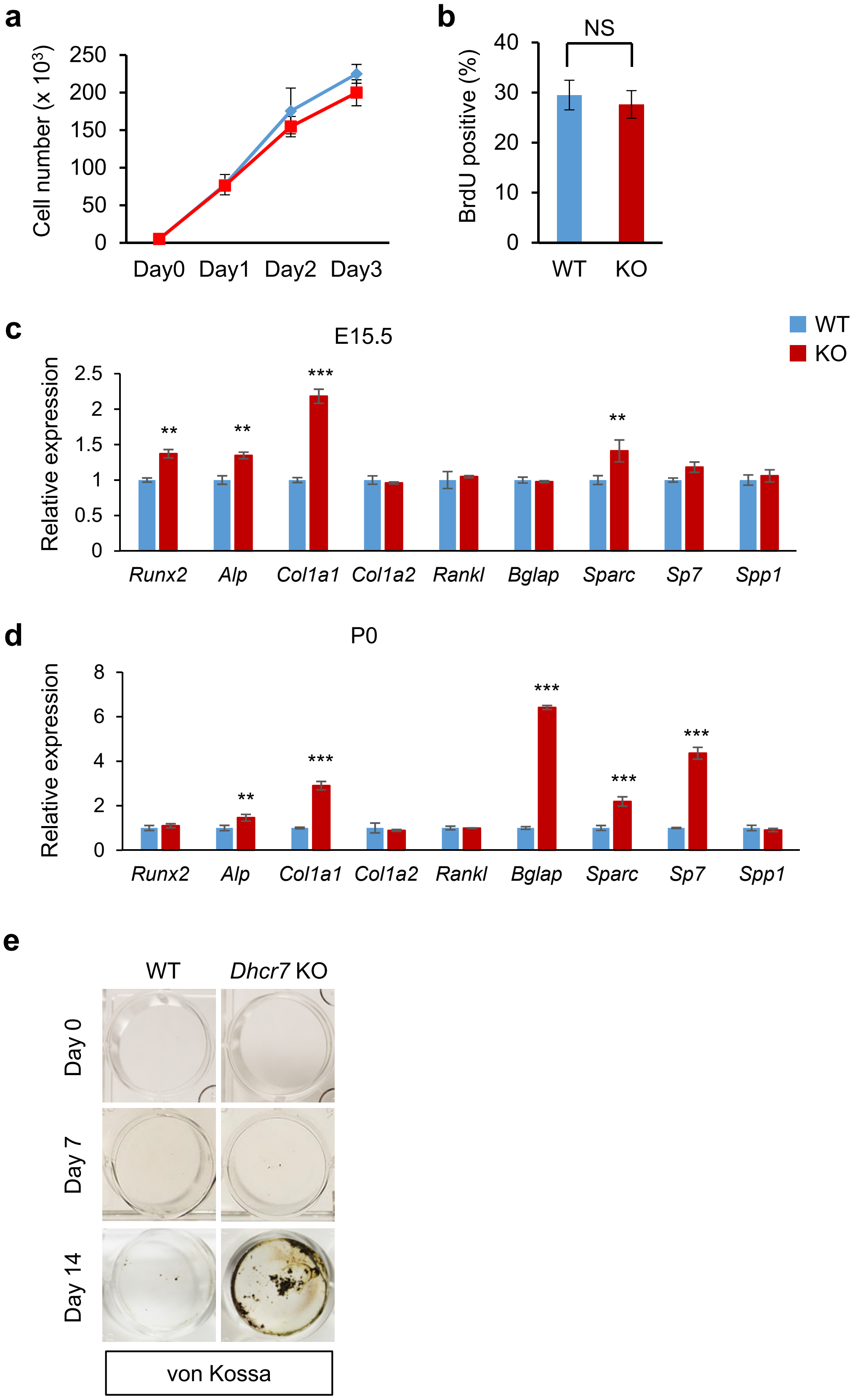
a
Cell number (x 10³)
250
200
150
100
50
0
Day0
Day1
Day2
Day3
b
NS
40
30
20
10
0
BrdU positive (%)
WT
KO
c
E15.5
WT
KO
Relative expression
2.5
2
1.5
1
0.5
0
**
**
***
**
Runx2
Alp
Col1a1
Col1a2
Rankl
Bglap
Sparc
Sp7
Spp1
d
P0
Relative expression
8
6
4
2
0
**
***
***
***
***
Runx2
Alp
Col1a1
Col1a2
Rankl
Bglap
Sparc
Sp7
Spp1
e
WT
Dhcr7 KO
Day 0
Day 7
Day 14
von Kossa

Figure S4

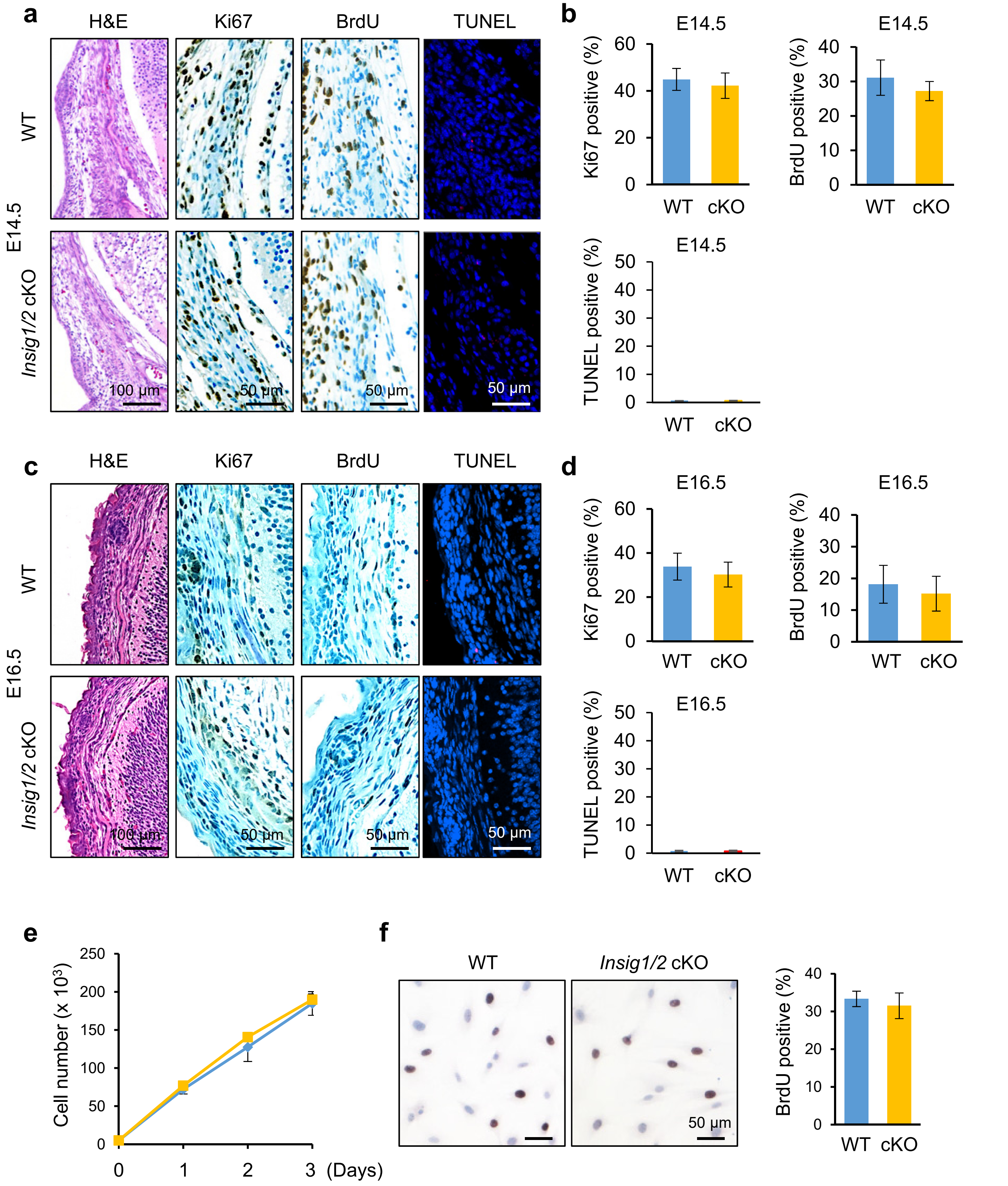

a
H&E
Ki67
BrdU
TUNEL
WT
E14.5
Insig1/2 cKO
100 µm
50 µm
50 µm
50 µm
b
E14.5
Ki67 positive (%)
WT
cKO
E14.5
BrdU positive (%)
WT
cKO
E14.5
TUNEL positive (%)
WT
cKO
c
H&E
Ki67
BrdU
TUNEL
WT
E16.5
Insig1/2 cKO
100 µm
50 µm
50 µm
50 µm
d
E16.5
Ki67 positive (%)
WT
cKO
E16.5
BrdU positive (%)
WT
cKO
E16.5
TUNEL positive (%)
WT
cKO
e
Cell number (x 10³)
(Days)
f
WT
Insig1/2 cKO
50 µm
BrdU positive (%)
WT
cKO

Figure S5

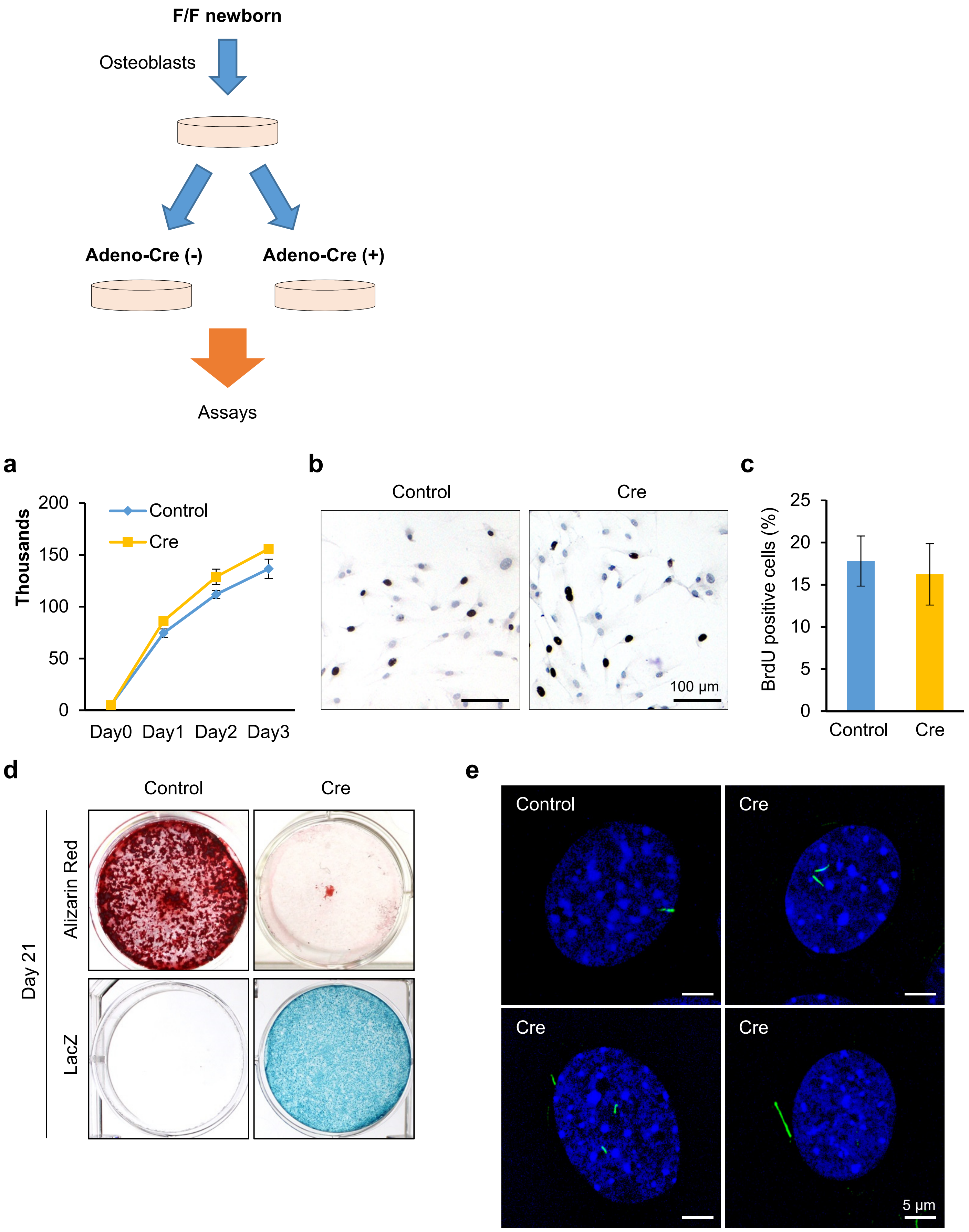

F/F newborn
Osteoblasts
Adeno-Cre (-)
Adeno-Cre (+)
Assays
a
Thousands
200
150
100
50
0
Control
Cre
Day0
Day1
Day2
Day3
b
Control
Cre
100 µm
c
BrdU positive cells (%)
25
20
15
10
5
0
Control
Cre
d
Control
Cre
Day 21
Alizarin Red
LacZ
e
Control
Cre
Cre
Cre
5 µm

Figure S6

a

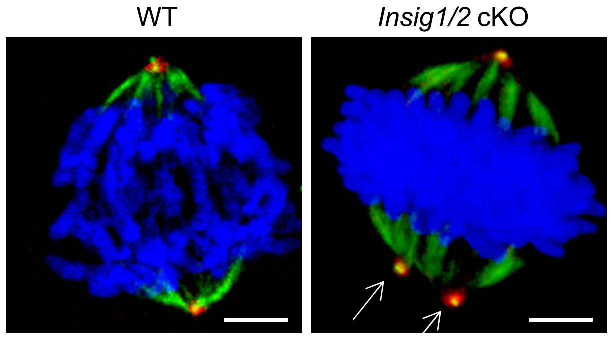
WT
Insig1/2 cKO

b

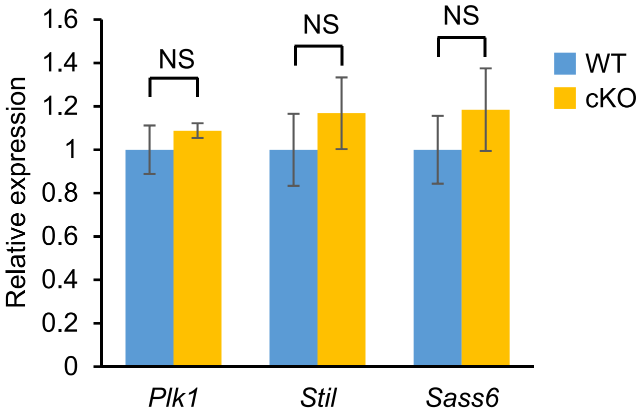
Relative expression
1.6
1.4
1.2
1
0.8
0.6
0.4
0.2
0
NS
NS
NS
WT
cKO
Plk1
Stil
Sass6

Figure S7

a

Col1a1 GLI – Binding Site (-68 to -60 bp)

```
Mouse          gacgggagtttctcctcgggacggagcag-gaggcacg--cggagtgaggccacgcatga
Rat            gacgggagtttcacctccggacggagcag-gaggcaca--cggagtgaggccacgcatga
Dog            gacgggagtttctcctcggggtcggagcaggaggcacgcggagtgtgaggccacgcatga
Horse          gacgggagtttcccctcggggtcggagcaggaggcacgcggagagtgaggccacgcatga
Macaque        gacgggagtttctccttggggtcggagcaggaggcacgcggagtgtgaggccacgcatga
Orangutan      gacgggagtttctcctcggggtcggagcaggaggcacgcggagtgtgaggccacgcatga
Human          gacgggagtttctcctcggggtcggagcaggaggcacgcggagtgtgaggccacgcatga
Chimpanzee     gacgggagtttctcctcggggtcggagcaggaggcacgcggagtgtgaggccacgcatga
               ************ ***  **   *      *******     * ****************
```

b

Col1a1 LEF1 – Binding Site 1(-29 to -25 bp)

```
Mus            gccgaagctaacccccccaccccagccgcaaagagtctacatgtctagggtctagacatgt
Rattus         gccgaagctaacccccccaccccagccgcaaagagtctacatgtctagggtctagacatgt
Dog            gccgacg-ctaaccccccaccccagccgcaaagagtctacatgtctaggatctagacatgt
Equus          gcggacg-ctaaccccccaccccagccgcaaagagtctacatgtctagggtctagacatgt
Rhesus         gccggacgctaacccccctccccagccgcaaagagtctacatgtctagggtctagacatgt
Orangutan      gcggacg-ctaacccccctccccagccgcaaagagtctacatgtctagggtctagacatgt
Homo           gcggacg-ctaacccccctccccagccacaaagagtctacatgtctagggtctagacatgt
Pan            gcggacg-ctaacccccctccccagccgcaaagagtctacatgtctagggtctagacatgt
               ** *      * ************************************************
```

Col1a1 LEF1 – Binding Site 2 (-484 to -480 bp)

```
Mus            caattattcctgacactgatcccttttgtagtgtttcccaactc-------agattctcag
Rattus         caattgtgcctgacactgaacccttttgtaatgttccccaactc-------agattcccaa
Dog            ggatcacccccattccttctacctctggaagattcccttcccagc--------tccccag
Equus          ccaattaccccaatccttccacctttggaaggctccctacccactcccc---------aa
Rhesus         ccaacacccccaacccttccacctttggaagtctccccacccagctc--------cccag
Orangutan      ccaacacccccaacccttccacctttggaagtctccccacccagctc--------cccag
Homo           ccaacacccccaacccttccacctttggaagtctccccacccagctccccagttccccag
Pan            ccaacacccccaacccttccacctttggaagtctccccacccagctccccagttccccag
                 *     **     **    *** ** *    * **   *                 *
```

Col1a1 LEF1 – Binding Site 3 (-1642 to -1638 bp)

```
Mus            --ttttccctttgccttcgttgcacaaaactagctcagggagggcgtgaagg--gggggg
Rattus         ttttttttctttgccttcgttgcacaaaactagctcagggagggcgtgaagg--g---gg
Dog            tttttttcctttgccttcgttgcacaaaaccagctggggggagggcgtggagaggggcggg
Equus          tttttttcctttgccttcgttgcacaaaaccagctggggggagggcgtggagaggggcggg
Rhesus         ttttcccctttgccttcgttgcacaaaaccagctggggggagggcgtggagaggggcggg
Orangutan      tttttcccctttgccttcgttgcacaaaaccagctggggggagggcgtggagaggggcggg
Homo           tttttcccctttgccttcgttgcacaaaaccagctggggggagggcgtggagaggggcggg
Pan            tttttcccctttgccttcgttgcacaaaaccagctggggggagggcgtggagaggggcggg
                 **    ********************** ****  *********** **   *   **
```

Col1a1 LEF1 – Binding Site 4 (-2713 to -2709 bp)

```
Mus            gggg--tggagatt-ctggcttttgctctccctacactccaagtgcctgacaaagcccta
Rattus         gggg--tggagatt-ctggcttttgctccccctacactccaagtgccggacaaagcccta
Dog            gtgggggggagattttggcttttgtttcccccccaactccaaatgccggactaagtctca
Equus          gg----aggagagtttgg--cttttg-ctccccccactccaaatgccggacaaagtctta
Rhesus         gg----aggagattttgg--cttttgctccccccctactttaagtgccggacaaagtctta
Orangutan      gg----aggagattttgg--cttttgctctcccccactttaagtgccggacaaagtctta
Homo           gg----aggagattttgg--cttttgctcccccccactttaagtgccggacaaagtctta
Pan            gg----aggagattttgg--cttttgctcccccccactttaagtgccggacaaagtctta
               *      ***** *   *   **       **   ***  ** **** *** *** *  *
```

Figure S8

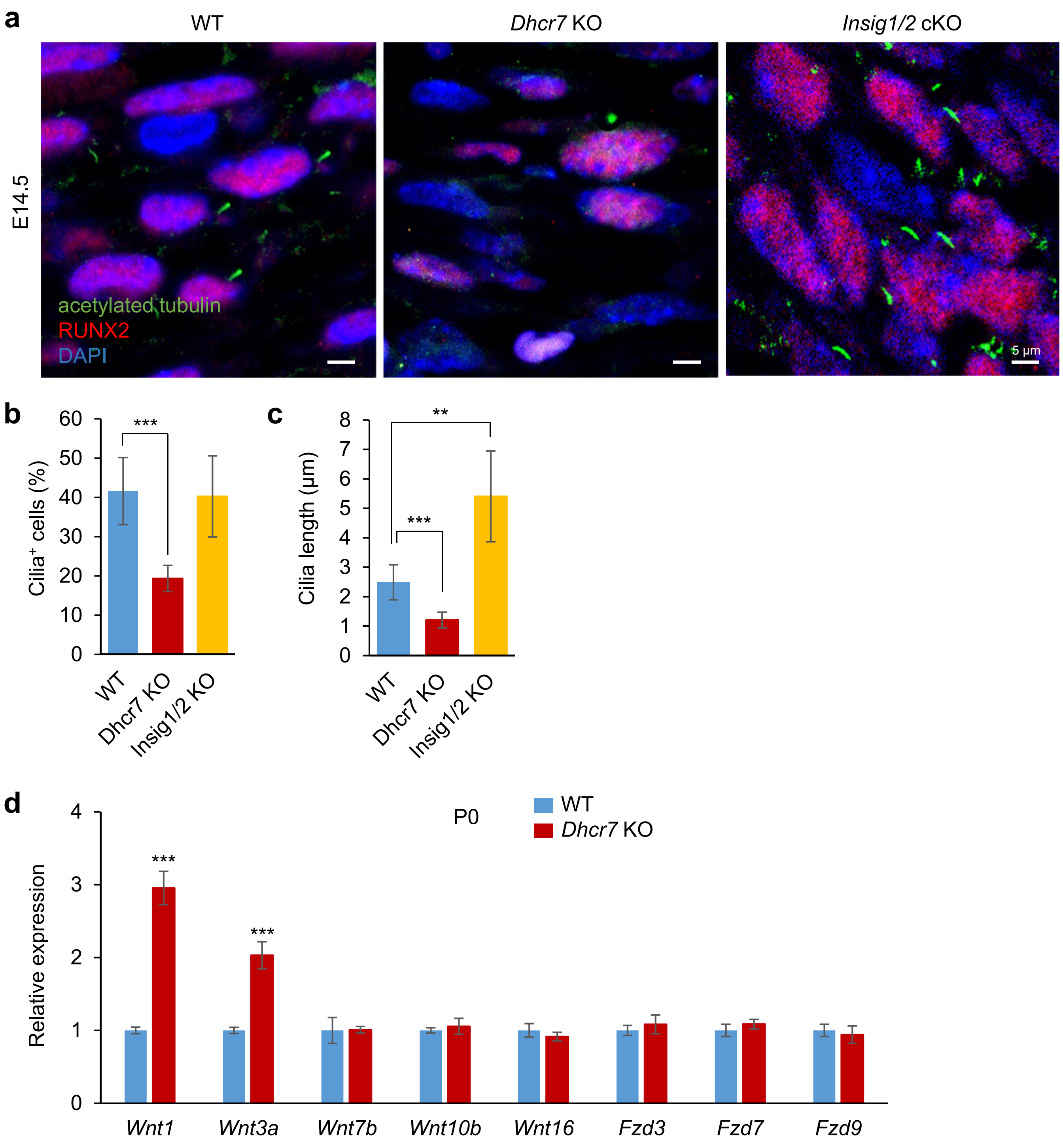
a
WT
Dhcr7 KO
Insig1/2 cKO
E14.5
acetylated tubulin
RUNX2
DAPI
5 µm
b
Cilia+ cells (%)
60
50
40
30
20
10
0
***
WT
Dhcr7 KO
Insig1/2 KO
c
Cilia length (µm)
8
7
6
5
4
3
2
1
0
**
***
WT
Dhcr7 KO
Insig1/2 KO
d
Relative expression
4
3
2
1
0
P0
WT
Dhcr7 KO
***
***
Wnt1
Wnt3a
Wnt7b
Wnt10b
Wnt16
Fzd3
Fzd7
Fzd9

Figure S9

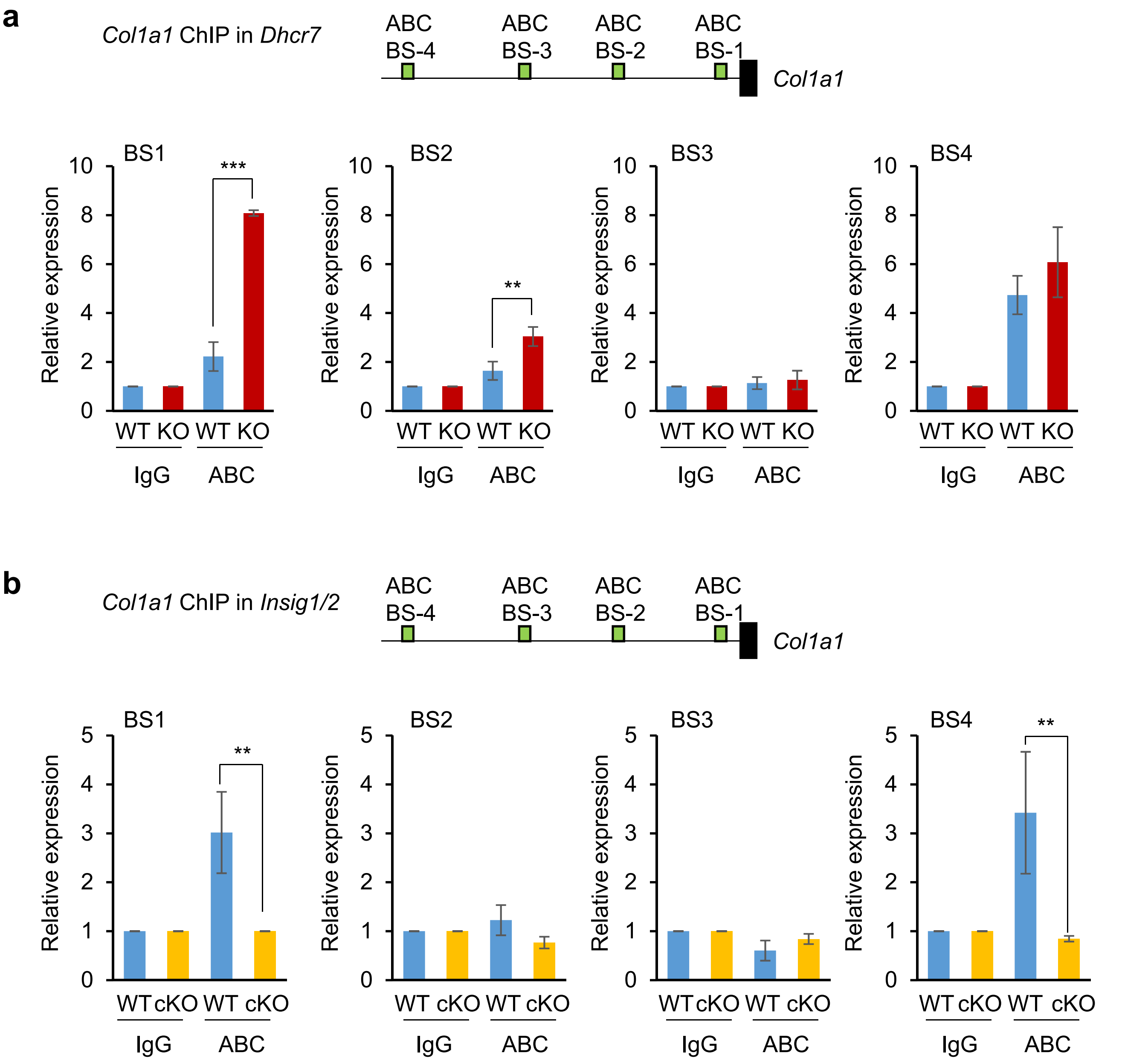
a
Col1a1 ChIP in Dhcr7
ABC BS-4
ABC BS-3
ABC BS-2
ABC BS-1
Col1a1
BS1
BS2
BS3
BS4
Relative expression
***
**
WT KO WT KO
IgG ABC
b
Col1a1 ChIP in Insig1/2
ABC BS-4
ABC BS-3
ABC BS-2
ABC BS-1
Col1a1
BS1
BS2
BS3
BS4
Relative expression
**
**
WT cKO WT cKO
IgG ABC
